# Quantitative prediction of intracellular dynamics and synaptic currents in a small neural circuit

**DOI:** 10.3389/fncom.2025.1515194

**Published:** 2025-09-01

**Authors:** Thiago B. Burghi, Kyra Schapiro, Maria Ivanova, Huaxinyu Wang, Eve Marder, Timothy O'Leary

**Affiliations:** ^1^Department of Engineering, University of Cambridge, Cambridge, United Kingdom; ^2^Volen National Center for Complex Systems, Brandeis University, Waltham, MA, United States

**Keywords:** central pattern generator, artificial neural networks, machine learning, neural circuits, electrophysiology, system identification, dynamic clamp

## Abstract

Fitting models to experimental intracellular data is challenging. While detailed conductance-based models are difficult to train, phenomenological statistical models often fail to capture the rich intrinsic dynamics of circuits such as central pattern generators (CPGs). A recent trend has been to employ tools from deep learning to obtain data-driven models that can quantitatively learn intracellular dynamics from experimental data. This paper addresses the general questions of modeling, training, and interpreting a large class of such models in the context of estimating the dynamics of a neural circuit. In particular, we use recently introduced Recurrent Mechanistic Models to predict the dynamics of a Half-Center Oscillator (HCO), a type of CPG. We construct the HCO by interconnecting two neurons in the Stomatogastric Ganglion using the dynamic clamp experimental protocol. This allows us to gather ground truth synaptic currents, which the model is able to predict–even though these currents are not used during training. We empirically assess the speed and performance of the training methods of teacher forcing, multiple shooting, and generalized teacher forcing, which we present in a unified fashion tailored to data-driven models with explicit membrane voltage variables. From a theoretical perspective, we show that a key contraction condition in data-driven dynamics guarantees the applicability of these training methods. We also show that this condition enables the derivation of data-driven frequency-dependent conductances, making it possible to infer the excitability profile of a real neuronal circuit using a trained model.

## 1 Introduction

Constraining data-driven predictive models of biological neuronal circuits presents significant challenges. Neuronal circuits exhibit substantial variability in connectivity strength and intrinsic physiological characteristics across specimens, and they operate under time-varying inputs and uncertainties (Schulz et al., 2006, 2007; Marder, 2012; Anwar et al., 2022). These challenges become particularly visible in circuits with complex intrinsic dynamics, such as Central Pattern Generators (CPGs), where the interplay of membrane currents across multiple timescales defines circuit-level properties (Goaillard et al., 2009; Grashow et al., 2009; Franci et al., 2017; Drion et al., 2018), but the same issues are also at play in circuits whose outputs are less well-defined.

Until recently, two broad classes of modeling approaches were used to predict the activity of neural circuits. The first uses phenomenological stochastic neural dynamics to predict spike timing, sometimes with remarkable success (Paninski et al., 2005; Gerstner and Naud, 2009; Gerstner et al., 2014; Pillow and Park, 2016). These so-called statistical models can potentially fit data very well, but their general-purpose integrate-and-fire structure limits mechanistic insight; in particular this approach cannot capture the rich intrinsic dynamics present in circuits such as CPGs. Additionally, with few exceptions (e.g., Latimer et al., 2019), statistical models fail to account for the mechanistic effects of membrane conductances, which limits their interpretability and prevents their use in closed-loop experiments involving dynamic clamp or neuromodulation (Sharp et al., 1993; Prinz et al., 2004a). The second class of modeling approaches attempts to constrain biophysically detailed models to experimental data, and a number of methods have been developed for that purpose (Huys et al., 2006; Abarbanel et al., 2009; Buhry et al., 2011; Meliza et al., 2014; Lueckmann et al., 2017; Abu-Hassan et al., 2019; Alonso and Marder, 2019; Taylor et al., 2020; Gonçalves et al., 2020; Wells et al., 2024). Detailed models are interpretable, but suffer from excessive complexity that makes it difficult to fit them to data efficiently (Geit et al., 2008). In addition, no consensus exists on how much detail can be safely ignored while still capturing essential dynamical properties and accounting for experimentally observed constraints (Almog and Korngreen, 2016). Existing methodologies require precise measurements and high computational resources, making it impractical to use within the time constraints of an experiment. Finally, these models may capture plausible parameter subspaces of misspecified models rather than the “true” parameters (Prinz et al., 2004b).

To address these challenges, a third class of modeling approaches has recently emerged that leverages the power of artificial neural networks (ANNs) and deep learning to efficiently capture the complexity of intracellular dynamics in a quantitative fashion (Brenner et al., 2022; Durstewitz et al., 2023; Aguiar et al., 2024; Burghi et al., 2025a). In such approaches, the vector field governing the membrane dynamics is parametrized using specific types of ANNs. The resulting models can therefore be interpreted as particular classes of Recurrent Neural Networks (RNNs) or Universal Differential Equations (Chen et al., 2018; Rackauckas et al., 2021), depending on whether time is treated as discrete or continuous. The goal of the present work is to understand how to efficiently train discrete-time models from this broad data-driven class, and how to interpret them in electrophysiological terms. To answer these questions, we combine theoretical analysis with empirical investigation. To pursue the latter, we work with the recently introduced class of Recurrent Mechanistic Models or RMMs (Burghi et al., 2025a), a data-driven architecture based on structured state space models (Gu et al., 2022) and ANNs which is geared for rapid estimation of intracellular neuronal dynamics.

Our empirical results show that RMMs can be trained to quantitatively predict the membrane voltage and synaptic currents in a non-trivial small neuronal circuit known as a Half-Center Oscillator (HCO). This circuit, composed of two reciprocally inhibitory neurons, autonomously generates anti-phasic bursting activity, and is the basis for oscillatory patterns in biology, such as the leech heartbeat (Calabrese et al., 1995; Wenning et al., 2004; Calabrese and Marder, 2024). Due to the complex intrinsic dynamics and extensive morphology of the cells involved in HCOs, fitting quantitatively predictive models to those circuits has historically been extremely challenging, even with access to high-performance computing and state-of-the-art numerical methods. While HCOs exist in nature, in this work we obtain one using the dynamic clamp technique (Sharp et al., 1993). This is done by creating artificial synapses between two Gastric Mill (GM) neurons in the Stomatogastric Ganglion of the crab *Cancer borealis*, similarly to previous work (Sharp et al., 1996; Grashow et al., 2009; Morozova et al., 2022). Defining the synaptic currents of the circuit ourselves provides ground truth connectivity data, which is used not for training but rather to assess the ability of RMMs to predict internal circuit connectivity. We show that RMMs can quantitatively predict synaptic currents from voltage measurements alone, that prediction accuracy depends on training algorithms, and that the accuracy improves when biophysical-like priors are introduced in the model.

A key contribution of this work is an empirical assessment of the speed and predictive performance of the training methods of teacher forcing (TF; Doya, 1992), multiple shooting (MS; Ribeiro et al., 2020b), and generalized teacher forcing (GTF; Doya, 1992; Abarbanel et al., 2009; Hess et al., 2023), which we present in a unified fashion tailored to models with an explicit membrane voltage variable (such as RMMs). From the theoretical perspective, we show how a key contraction property (Lohmiller and Slotine, 1998) of the internal neuronal dynamics guarantees the well-posedness of all three aforementioned training methods. The contraction property, which can be verified in a data-driven model by means of linear matrix inequalities, has been exploited in control theory for system identification (Manchester et al., 2021), for robustly learning data-driven models (Revay et al., 2024), and for estimating conductance-based models in real-time (Burghi and Sepulchre, 2024).

We also show that the contraction property enables a rigorous analysis of RNN-based data-driven models in terms of *frequency-dependent conductances*, which generalize the familiar neuronal input conductance, and are related to the sensitivity functions of control theory (Franci et al., 2019). Using RMMs, we obtain and interpret data-driven frequency-dependent conductances of the experimental HCO circuit.

In studying RNN-based methods using RMMs as a representative example, our results also extend the results of Burghi et al. (2025a), which focused on single-compartment dynamics. We find that our flexible, lightweight RMM approach enables us to build a predictive model of a complex neuronal circuit within the timeframe of an experiment. This paves the way for closed-loop, model-based manipulations of neural activity. Crucially, the model structure provides interpretable measurements of the circuit in quasi-real-time, facilitating novel types of diagnostic and scientific experiments on living neural circuits.

## 2 Results

Our results address three key questions related to data-driven neuronal circuit models where the membrane dynamics of individual neurons is parametrized with artificial neural networks (ANNs). First, *how to best learn such models efficiently*, that is, how to formulate an optimization algorithm capable of rapidly delivering good predictive models of the real neuronal circuit activity. Second, *whether such models can accurately predict unmeasured synaptic currents* inside a neuronal circuit. Third, *how to interpret such a model in terms of biophysically meaningful quantities*.

Our results are relevant to a wide class of data-driven models with ANN-based membrane dynamics. To facilitate working with sampled data, models are formulated in discrete time. The *input-output data* used to train a model is given by a sequence {*v*_*t*_, *u*_*t*_} of vector-valued measured intracellular membrane voltages *v*_*t*_ and vector-valued measured injected electrical currents *u*_*t*_, observed at discrete time points *t*∈ℕ. The dynamics of a circuit model with *n*≥1 neurons is described by the discrete-time state-space model


(1a) $$\begin{array}{l} C\frac{{\hat{v}}_{t+1}-{\hat{v}}_{t}}{\delta }=-h_{\theta }\left({\hat{v}}_{t},x_{t}\right)+u_{t} \end{array}$$

(1b) $$\begin{array}{l} x_{t+1}=f_{\eta }\left({\hat{v}}_{t},x_{t}\right) \end{array}$$

where ${\hat{v}}_{t}=\left({\hat{v}}_{1,t},\ldots ,{\hat{v}}_{n,t}\right)$ is a state vector gathering all predicted membrane voltages, *u*_*t*_ = (*u*_1, *t*_, …, *u*_*n, t*_) is an input vector gathering all (measured) injected currents, and *x*_*t*_ is a state vector gathering internal (also known as hidden or latent) state variables. The system is parametrized by a fixed sampling period δ>0, a learnable diagonal membrane capacitance matrix *C*, and learnable piecewise differentiable mappings *f*_η_ and *h*_θ_, whose parameters are the vectors θ and η, respectively. Notice that *C* can in principle be estimated separately through voltage-clamp (Taylor, 2012); this is not pursued in our paper.

The motivation behind Equation 1 comes from the simplest of all mechanistic constraints: the membrane dynamics is assumed to be reasonably modeled by Kirchoff's laws. Equation 1 thus describes the balance between a capacitive current


$$\begin{array}{l} C\overset{\cdot }{\hat{v}}\approx C\delta^{-1}\left({\hat{v}}_{t+1}-{\hat{v}}_{t}\right), \end{array}$$

an injected current *u*, and a number of ionic and synaptic currents whose sum total is given by the output of *h*_θ_. We are interested in models where the functions *h*_θ_ and *f*_η_ are constructed with ANNs. Models that follow this idea are Piecewise Linear Recurrent Neural Networks (Durstewitz et al., 2023), Recurrent Mechanistic Models (Burghi et al., 2025a), and traditional Recurrent Neural Networks (RNNs) where some of the states are explicitly identified with membrane voltages. All such models can be written in the form Equation 1 by re-labeling their states and re-parametrizing learnable variables. Notice that such models can be formulated in continuous-time following the frameworks of Neural (Chen et al., 2018) and Universal (Rackauckas et al., 2021) ODEs. Discretized conductance-based models in the Hodgkin-Huxley style (Izhikevich, 2007; Hodgkin and Huxley, 1952) fit the structure of Equation 1, but do not traditionally incorporate ANNs. The dynamics of HH-type ionic currents are instead based on detailed modeling assumptions informed by voltage-clamp experiments. Abandoning the HH formalism raises the question of how to gain relevant insights about the physiology of a neuronal circuit. This is addressed at the end of Results.

Over the next sections we describe different training methods used for inferring the dynamics of an arbitrary neural circuit using models of the form Equation 1: teacher forcing (TF), multiple shooting (MS), and generalized teacher forcing (GTF). Our experimental results assess the efficiency of TF, MS, and GTF by working with a Half-Center Oscillator circuit, which introduced next.

### 2.1 Model of a half-center oscillator

**Figure 1:**
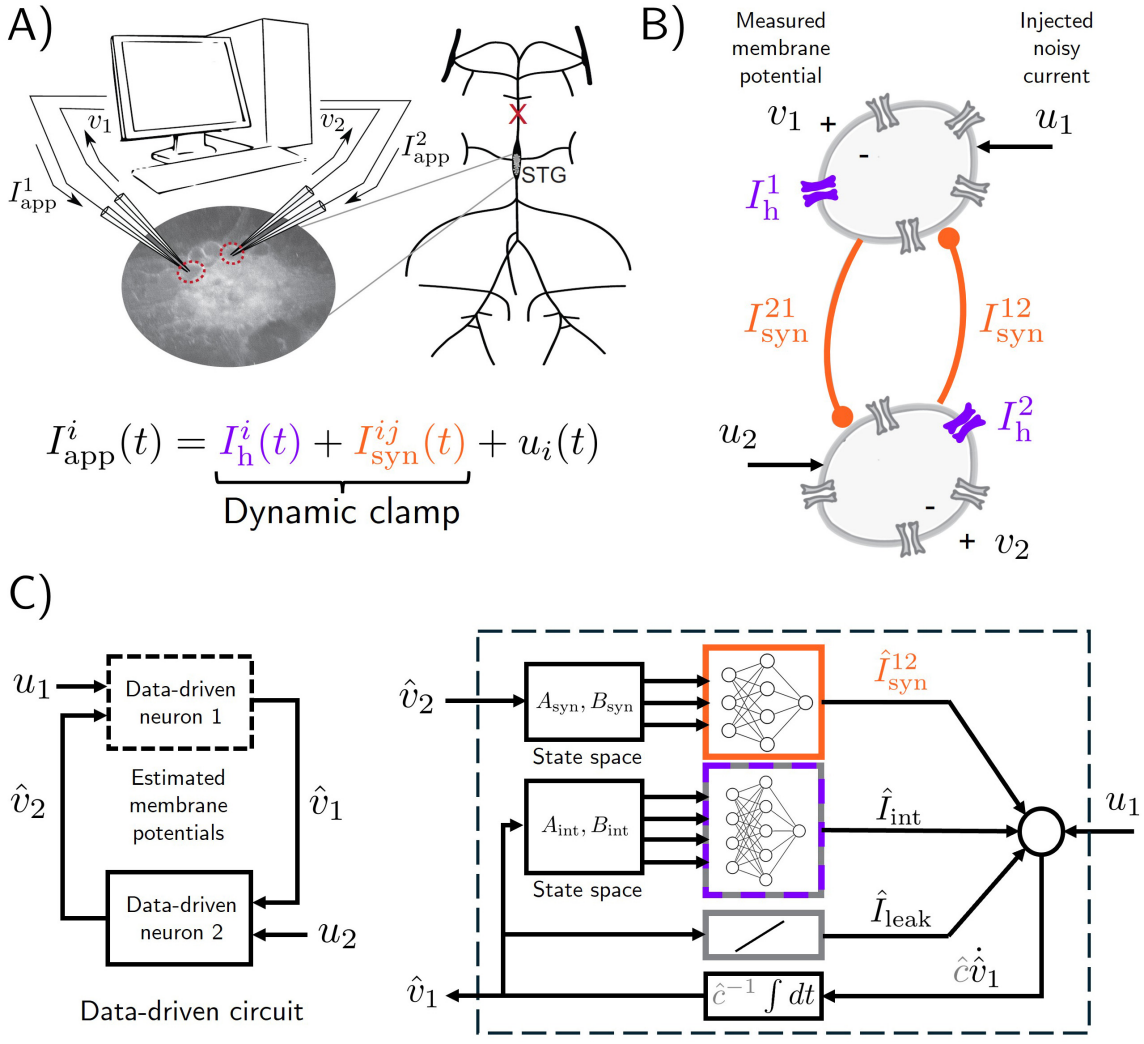
Predicting the intracellular membrane voltages and synaptic currents of a real neuronal circuit. **(A)** we estimate the dynamics of a Half-Center Oscillator (HCO) constructed experimentally with dynamic clamp. Two neurons from the Stomatogastric Ganglion of *Cancer borealis* are given virtual hyperpolarization-induced intrinsic currents $I_{\text{h}}^{i}$ and virtual inhibitory synaptic currents $I_{\text{syn}}^{ij}$, which are implemented via dynamic clamp Prinz et al. (2004a) (see Methods). In addition to the dynamic clamp currents, a noisy current *u*_*i*_ is also injected in each neuron. Illustration adapted from Morozova et al. (2022). **(B)** A diagram of the resulting HCO. Dynamic clamp currents are pictured in color; naturally occurring ionic currents are pictured in gray. **(C)** Conceptual diagram of a data-driven model used to learn the HCO dynamics, with a detailed diagram of neuron 1 on the right. The membrane dynamics of each of the neurons in the HCO is modeled using a combination of linear state-space models and multi-layer perceptrons, following the *Recurrent Mechanistc Model* paradigm of Burghi et al. (2025a) (see Methods for the specific model used in this figure). The data used to learn the model are the measured membrane voltages *v*_1_ and *v*_2_ and the noisy injected currents *u*_1_ and *u*_2_.

Concretely, we consider the neuronal circuit given by the experimental preparation illustrated in Figure 1A. The two-neuron circuit, which is commonly known as a Half-Center Oscillator (HCO), consists of two cells with measured membrane voltages *v*_1, *t*_ and *v*_2, *t*_, interconnected via inhibitory synapses. In naturally occurring HCOs, synaptic currents are typically not measured due to the difficulty of obtaining them while the circuit is intact. For this reason, and to provide ground truth data against which synaptic current predictions of data-driven models can be validated, the HCO preparation used in this paper is created artificially with dynamic clamp (Sharp et al., 1993). Dynamic clamp is used to inject two electrical current components into the membrane of each neuron: a virtual inhibitory synaptic current $I_{\text{syn},\text{t}}^{ij}$ and a virtual hyperpolarization-induced current $I_{\text{h},\text{t}}^{i}$. In addition to dynamic clamp currents, each neuron is also excited with a noisy injected current *u*_*i, t*_, which is used to explore the dynamics of the circuit and ensure that the data is informative enough for the purposes of data-driven modeling. See *applied current* in Methods for details. The particular HCO used in this paper is created using two cells in the Stomatogasteric Ganglion of the crab, and the procedure for forming this circuit with dynamic clamp is can be found in (Morozova et al. 2022). A diagram of the resulting HCO is shown in Figure 1B, where artificial circuit elements introduced by dynamic clamp are depicted in color, while biological elements are depicted in gray.

To estimate the HCO dynamics, we use the Recurrent Mechanistic Model (RMM) framework of (Burghi et al. 2025a). The RMM used throughout this paper is depicted in Figure 1C. It is described in succinct form by


(2) $$\begin{array}{l} C\frac{{\hat{v}}_{t+1}-{\hat{v}}_{t}}{\delta }=-\text{ml}{\text{p}}_{\text{θ}}\left({\hat{v}}_{t},x_{t}\right)+u_{t}\\ \;x_{t+1}=Ax_{t}+B{\hat{v}}_{t} \end{array}$$

where *A* and *B* are matrices of the appropriate dimensions, and $\text{ml}{\text{p}}_{\theta }\left({\hat{v}}_{t},x_{t}\right)$ is a multi-layer perceptron (MLP) whose inputs are given by ${\hat{v}}_{t}=\left({\hat{v}}_{1,t},{\hat{v}}_{2,t}\right)$ and *x*_*t*_. For a description of the model in terms of individual circuit neurons, see *modeling details* in Methods. It can be seen that the RMM in Equation 2 greatly constrains the more general formulation (Equation 1) by imposing a linear state-space dynamics of the internal states *x*_*t*_, and by imposing a feed-forward artificial neural network structure on *h*_θ_.

Training the RMM means finding good values for *C* and θ so that the model is able to predict the response of the HCO to the injected noisy currents *u*_1, *t*_ and *u*_2, *t*_. For our purposes, the HCO response consists of the measured voltages *v*_1, *t*_ and *v*_2, *t*_ (which are used during training), and the synaptic currents $I_{\text{syn},\text{t}}^{12}$ and $I_{\text{syn},\text{t}}^{21}$ (which are not). Training the RMM allows us to assess various training algorithms that can also be used to train other types of data-driven models. RMMs are convenient for that purpose since they can be rapidly trained. In the results below we have intentionally limited the size of the RMM, as well as the amount of data used in training, so that a meaningful comparison between learning methods could be pursued.

### 2.2 Training data-driven models: a unified perspective

Training data-driven models of the form Equation 1 to learn complex dynamics such as spiking and bursting is in general not trivial. This is because the *forward dynamics* of neuronal models, i.e. the mapping from applied current (*u*_*t*_) to predicted membrane voltage (${\hat{v}}_{t}$), is highly sensitive to changes in the model's parameters. The sensitivity leads to the problem of *exploding gradients* (Pascanu et al., 2013) when one approaches the learning problem naively. To avoid exploding gradients, the standard approach for training neuronal models is to exploit the structure given by Equation 1 and employ some variation or generalization of the method of *teacher forcing* (Doya, 1992). We approach the problem of training neuronal data-driven models from a unified perspective, relating the methods of *teacher forcing, multiple shooting*, and *generalized teacher forcing* to each other. Throughout this section, we consider an exponential contraction condition (Lohmiller and Slotine, 1998) on the internal dynamics of the generic model (Equation 1), which we show to be sufficient for the applicability of these training methods. To make this paper self-contained, this condition is recapped in the Methods section. In what follows, *v*_*t*_ and *u*_*t*_ denote the experimentally recorded voltage and applied current, respectively.

#### 2.2.1 Recurrent and feed-forward teacher forcing (TF)

The idea in the teacher forcing method is to train a model of the form Equation 1 by solving the optimization problem


(3a) $$\begin{array}{ll} \underset{\theta ,\eta ,\xi ,C}{\min}\; & \frac{1}{N}\overset{N-1}{\underset{t=0}{\sum }}||v_{t+1}-{\overset{⌄}{v}}_{t+1}{||}^{2}+r_{\rho }\left(\theta ,\eta \right) \end{array}$$

(3b) $$\begin{array}{ll} \text{subject to } & {\overset{⌄}{v}}_{t+1}=v_{t}+\delta C^{-1}\left(-h_{\theta }\left(v_{t},{\overset{⌄}{x}}_{t}\right)+u_{t}\right) \end{array}$$

(3c) $$\begin{array}{ll} \; & {\overset{⌄}{x}}_{t+1}=f_{\eta }\left(v_{t},{\overset{⌄}{x}}_{t}\right),\;{\overset{⌄}{x}}_{0}=\xi \end{array}$$

where *r*_ρ_(θ, η) is a regularization term, and ρ is a positive hyperparameter. We shall use $r_{\rho }\left(\theta ,\eta \right)=\rho ||\theta {||}^{2}+\rho ||\eta {||}^{2}$ throughout the paper. Equation 3 corresponds to minimizing the squared one-step-ahead voltage prediction error, with the one-step-ahead prediction ${\hat{v}}_{t+1}$ obtained by *forcing* the vector field of the model (Equation 1) with the “teacher” signal *v*_*t*_. Notice that it is crucial to distinguish the predictions used for training (${\hat{v}}_{t}$), computed with Equations 3b–3c, from the predictions of the simulated model (${\hat{v}}_{t}$), computed with Equation 1. Teacher forcing uses the former as a means to improve the latter, and it should be emphasized that good ${\hat{v}}_{t}$ predictions do not necessarily imply good ${\hat{v}}_{t}$ predictions. Letting L(θ,η,ξ,C) denote the overall loss function of Equation 3, obtained by simulating Equation 3b–3c and substituting the result in Equation 3a, the problem is usually solved using some variant of the basic gradient descent (GD) method, which in our context can be written as


(4) $$\begin{array}{l} \theta_{k+1}=\theta_{k}-\beta \nabla_{\theta }L\left(\theta_{k},\eta_{k},\xi_{k}\right) \end{array}$$

for θ, and analogously for η, ξ, and *C* (here, β is the step size and *k* = 0, 1, 2, … are the training epochs). In non-convex problems, two conditions are necessary for finding good data-driven model parameters with gradient descent or any of its enhanced versions, such as ADAM (Kingma and Ba, 2017). First, one must choose a reasonable set of initial training parameters ξ_0_, θ_0_, η_0_, and *C*_0_. Second, one must ensure that the gradients in $\nabla L\left(\theta_{k},\eta_{k},\xi_{k}\right)$ remain well-behaved throughout training.

TF as stated in Equation 3 involves temporal *recurrence* in the dynamic constraints (Equation 3b–3c). Consequently, computing the gradients in Equation 4 requires *backpropagating through time*, which might be computationally costly depending on the length of the dataset. Recurrence can be eliminated from the problem by foregoing the learning of the initial state ξ and the internal dynamics parameters η. If both ξ and η remain constant during training, TF becomes a *feed-forward* learning problem. One can then divide training into two steps: first, simulate


(5) $$\begin{array}{l} {\overset{⌄}{x}}_{t+1}^{0}=f_{\eta_{0}}\left(v_{t},{\overset{⌄}{x}}_{t}^{0}\right),\;{\overset{⌄}{x}}_{0}^{0}=\xi_{0} \end{array}$$

for fixed η_0_ and ξ_0_, then, solve the (unconstrained) problem


(6) $$\begin{array}{l} \underset{\theta ,C}{\min}\;\frac{1}{N}\overset{N-1}{\underset{t=0}{\sum }}||v_{t+1}-v_{t}-\delta C^{-1}\left(-h_{\theta }\left(v_{t},{\overset{⌄}{x}}_{t}^{0}\right)+u_{t}\right){||}^{2}+r_{\rho }\left(\theta \right) \end{array}$$

using the fixed time series of internal states ${\overset{⌄}{x}}_{t}^{0}$. We say Equation 6 is a feed-forward problem because it reduces to training the mapping $C^{-1}\left(h_{\theta }\left(v_{t},{\overset{⌄}{x}}_{t}^{0}\right)+u_{t}\right)$, which in data-driven models such as Equation 2 will usually contain feed-forward ANNs. Solving this problem is significantly faster than solving its recurrent counterpart (Equation 3), since backpropagating through time is no longer required—backpropagation is limited to the layers of *h*_θ_. This idea, which we call *feed-forward TF*, has previously been used to train conductance-based models by taking existing ion channel models, and training the voltage dynamics with fixed ion channel kinetics (Huys et al., 2006).

An exponential contraction condition of the internal states (see *contraction theory* in Methods) guarantees that both recurrent and feed-forward versions of TF are well-posed. In the former, it precludes exploding gradients, since one can show that if the internal dynamics is contracting, uniformly in *v* and η, then the loss gradients ∇L remain bounded. In addition, in both versions of TF above, contraction helps to improve learning performance by using *warmed up initial conditions* ξ_0_ (see *training details* in Methods).

**Figure 2:**
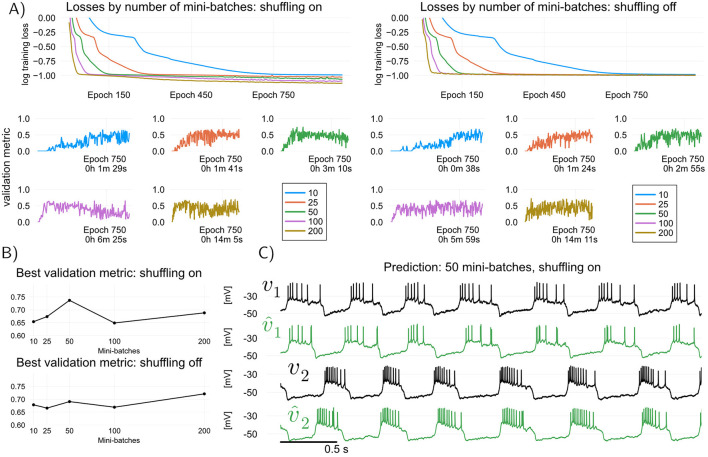
Effect of number of mini-batches and dataset shuffling in feed-forward teacher forcing (TF). To assess predictive performance of trained models, we use a modified cosine similarity metric ranging from 0 (no spike alignment) to 1 (perfect spike alignment), see *training details* in Methods. **(A)** TF training losses over epochs, with shuffling disabled and enabled, for varying numbers of mini-batches. In both cases, increasing the number of mini-batches (i.e., reducing batch size) generally improves convergence speed of training loss, since more gradient descent steps are taken per epoch. Smaller batch sizes (more batches) accelerate convergence but overfit more quickly, as shown by the drop in validation metric after an initial rise. In contrast, larger batch sizes (fewer batches) converge more slowly but yield stabler validation results from where to choose a suitable model (models are saved every 5 epochs). Overfitting in TF is an expected consequence of the difference between training and validation objectives. A first sharp drop in TF losses leads to a sharp increase in predictive performance, while a second sharp drop leads to overfitting. Shuffling leads to a significant increase in convergence speed and quicker overfitting. **(B)** Best validation metric attained during training for each mini-batch configuration, comparing shuffled (bottom) and non-shuffled (top) datasets. **(C)** Target voltage traces (held-out data, in black) and predicted voltage traces (in green) obtained by simulating the best HCO data-driven model (RMM) after TF training (mini-batch size: 50; shuffling enabled).

In feed-forward TF, two tricks can be employed to speed up training and yield models that generalize better on unseen data. First, one can use *mini-batching* (Li et al., 2014) to perform GD parameter updates that only use part of the dataset. This is done by taking gradients of partial sums of the total loss function (Equation 6), resulting in better exploration of the loss landscape. Second, one can *shuffle* (Meng et al., 2019) the dataset (that is, randomly permute the data points) *before* partitioning the summation in the cost (Equation 6) into mini-batches; this can sometimes lead to better generalization. In Figure 2 we apply feed-forward TF to the RMM data-driven model of the HCO, and compare the effect of mini-batch size and shuffling on the predictive power of trained models. Models are obtained with ρ = 5 × 10^−8^, which was selected after a simple grid search over regularization constants. Validation traces are shown in terms of a modified cosine similarity metric which is applied after filtering and smoothing out the spikes in the dataset, see Methods. It is seen that in general, increasing the number of batches in a dataset (decreasing the mini-batch size) is beneficial up to a point. It can also be seen that shuffling the dataset during training results in faster improvements on the validation metric, but quicker overfitting due to the significant increase in convergence speed.

The most distinctive aspect of TF training is its susceptibility to overfitting, which can be observed in Figure 2A and is more prominent when shuffling is enabled. This is because the TF problem in Equation 3a or 6 minimizes discrepancies in the map (the right-hand side) of the voltage dynamics in Equation 1, whereas the validation metric assesses discrepancies in the simulated voltage trace. By saving “snapshots” of trained models during training with feed-forward TF, overfitting can be avoided, and reasonably good models obtained in a short amount of time. This is shown in Figure 2B, where the performance of the best model over the course of training for for different number of mini-batches is plotted. As Figure 2C shows, a validation metric of around 0.73 results in visually adequate good predictions, with a few intra-burst spikes of one of the HCO neurons being missed over a few bursts. Increasing model size, choosing a better exploratory injected current *u*_*t*_, and conducting an extensive hyperparameter search are all strategies that can be pursued to improve models obtained with TF.

#### 2.2.2 Multiple shooting trades off speed for performance

Teacher forcing is significantly limited by the quality of the data. This becomes evident when one interprets the loss (Equation 6) in terms of a target voltage difference *v*_*t*+1_−*v*_*t*_. This difference is a high-pass filtering operation that amplifies the variance in measurement noise, which is ubiquitously present in neurophysiological applications. To deal with this problem, one must take into account the recurrence between voltage and internal dynamics, which is accomplished by generalizing teacher forcing. One way to do so is to employ *multiple shooting*, which is based on a classical method for finding numerical solutions of differential Equations (Ribeiro et al., 2020b).

**Figure 3:**
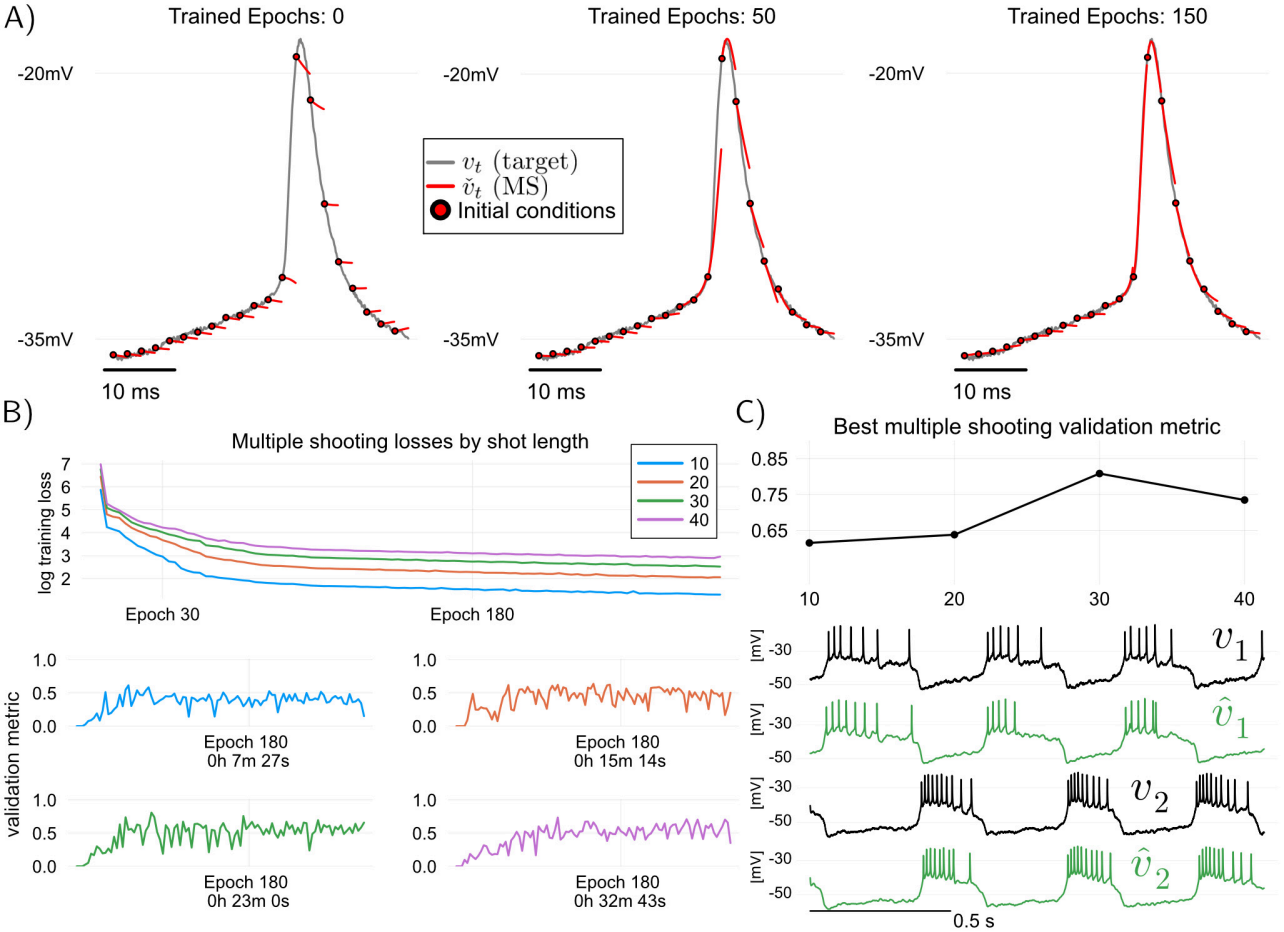
Training and validating the RMM (Equation 2) using multiple shooting, which trades off training speed for predictive performance. Here, modified angular separation in the interval [0, 1] is used as validation metric, see *training details* in Methods **(A)** Illustration of predictive “shots” during multiple shooting training. As training progresses, initial shot conditions as well as model parameters are learned. **(B)** Multiple shooting training loss (top) and modified angular separation (validation metric) of trained models, plotted for four different shot sizes. Increasing the shot length, training losses and training time increase, but predictive power of trained models (as measured by angular separation) may also increase. One advantage of Multiple shooting over Teacher Forcing is the relatively stable and monotonic increase in validation performance as a function of training epoch. **(C)** Maximum validation metric attained for each of the shot sizes plotted, and voltage traces of validation target (held-off data) and predictions of the best model (shot size of 30 samples). All models were trained starting from random MLP weights (Methods).

Thea idea in multiple shooting is to divide a dataset of length *N* into *N*_*s*_ = ⌊*N*/*s*⌋ intervals or “shots” of size *s*>1, and then train the model by simulating it in parallel over each of the shots separately (we use ⌊·⌋ for the round-down operator). Figure 3A illustrates this idea. Mathematically, when applied to models of the form Equation 1, multiple shooting takes the form


(7a) $$\begin{array}{ll} \underset{\theta ,\eta ,\xi^{\left(n\right)},\upsilon^{\left(n\right)},C}{\min}\; & \frac{1}{sN_{s}}\overset{N_{s}-1}{\underset{n=0}{\sum }}\overset{s-1}{\underset{t=0}{\sum }}||v_{t+ns}-{\overset{⌄}{v}}_{t}^{\left(n\right)}{||}^{2}+r_{\rho }\left(\theta ,\eta \right)\\ \; & +\rho_{v}||{\overset{⌄}{v}}_{s}^{\left(n\right)}-\upsilon^{\left(n+1\right)}{||}^{2}+\rho_{x}||{\overset{⌄}{x}}_{s}^{\left(n\right)}-\xi^{\left(n+1\right)}{||}_{D}^{2} \end{array}$$

(7b) $$\begin{array}{ll} \text{subject to } & {\overset{⌄}{v}}_{t+1}^{\left(n\right)}={\overset{⌄}{v}}_{t}^{\left(n\right)}+\delta C^{-1}\left(-h_{\theta }\left({\overset{⌄}{v}}_{t}^{\left(n\right)},{\overset{⌄}{x}}_{t}^{\left(n\right)}\right)+u_{t+ns}\right),\\ \; & {\overset{⌄}{v}}_{0}^{\left(n\right)}=\upsilon^{\left(n\right)} \end{array}$$

(7c) $$\begin{array}{ll} \; & {\overset{⌄}{x}}_{t+1}^{\left(n\right)}=f_{\eta }\left({\overset{⌄}{v}}_{t}^{\left(n\right)},{\overset{⌄}{x}}_{t}^{\left(n\right)}\right),\;{\overset{⌄}{x}}_{0}^{\left(n\right)}=\xi^{\left(n\right)} \end{array}$$

where Equations 7b–7c describe the *N*_*s*_ simulations involved in the learning algorithm, run for *t* = 0, 1, …, *s*−1. In Equation 7a, the initial conditions of the voltage and internal states in each shot, υ^(*n*)^ and ξ^(*n*)^ respectively, must be learned. Assuming that the contraction condition holds, initial GD conditions $\xi_{0}^{\left(n\right)}$ can be chosen effectively by warming up the model (see Methods); for voltage, one can simply take $\upsilon_{0}^{\left(n\right)}=v_{ns}$. When *s* = *N*, multiple shooting becomes equivalent to minimizing the discrepancy between the measured *v* and the simulated $\hat{v}$ from Equation 1 over the entire dataset, which requires backpropagating through many bursts and spikes, and likely leads to exploding gradients. For *s* = 1, and fixed $\upsilon^{\left(n\right)}=v_{n}$, multiple shooting becomes equivalent to the dynamic TF problem (Equation 3a). Hence multiple shooting provides a means to trade off the speed of feed-forward TF for the enhanced predictive power of models trained with backpropagation through time, while avoiding exploding gradients due to the limited simulation horizon of each shot (Ribeiro et al., 2020a). It is worth noticing the term involving ρ_*x*_>0 in the cost function (Equation 7a): it is a regularizing term that attempts to drive the final internal states in each shot toward the initial states of the next shot.

When using multiple shooting in practice, it is not obvious how to choose values for the hyperparameters *s* (shot length), ρ (parameter regularization) and ρ_*v*_, ρ_*x*_ (final state regularization). Such questions are nearly impossible to answer theoretically, and one must resort to using hyperparameters that are found empirically to be the best for fitting a given neuronal circuit. Fixing the regularization constants ρ = 5 × 10^−9^, and ρ_*v*_ = ρ_*x*_ = 500, Figure 3 illustrates the effect of increasing the shot length *s* on training and validation with multiple shooting. In Figure 3B, It can be seen that by increasing the shot length *s*, predictive power of the models may increase, but this comes at the cost of increased training times. In particular, for the hyperparameters tested in this paper, MS results in a better model than TF (modified cosine similarity of 0.8 for MS versus 0.73 for TF, cf Figures 2, 3). One advantage of multiple shooting over TF is the fact that increases in the validation metric are relatively monotonic as a function of epochs. This means that, to obtain a good model, one can save “model snapshots” at a lower rate during training.

#### 2.2.3 Generalized teacher forcing yields a filter for the membrane dynamics

Generalized teacher forcing (GTF), first suggested by Doya (1992) and recently revisited in Hess et al. (2023), extends TF by forcing the model dynamics with a convex combination of the data and the model's outputs. GTF was formulated with the same goal as multiple shooting: to avoid exploding gradients when learning recurrent models. When specialized to neuronal data-driven models, GTF can be formulated by replacing the voltage data *v*_*t*_ in the TF problem (Equation 3) by a convex combination of *v*_*t*_ and voltage predictions ${\overset{⌄}{v}}_{t}$. GTF has also been proposed, although not under that name, to learn continuous-time conductance-based models in Abarbanel et al. (2009) (see also Brookings et al., 2014). To learn the dynamics of discrete-time neuronal models, the GTF formulations of both Doya (1992) and Abarbanel et al. (2009) can be reconciled through the following problem:


(8a) $$\begin{array}{ll} \underset{\theta ,\eta ,\xi }{\min}\; & \frac{1}{N}\overset{N-1}{\underset{t=0}{\sum }}||v_{t}-{\overset{⌄}{v}}_{t}{||}^{2}+r_{\rho }\left(\theta ,\eta \right) \end{array}$$

(8b) $$\begin{array}{ll} \text{subject to } & {\overset{⌄}{v}}_{t+1}^{-}={\overset{⌄}{v}}_{t}+\delta C^{-1}\left(-h_{\theta }\left({\overset{⌄}{v}}_{t},{\overset{⌄}{x}}_{t}\right)+u_{t}\right) \end{array}$$

(8c) $$\begin{array}{ll} \; & {\overset{⌄}{x}}_{t+1}=f_{\eta }\left({\overset{⌄}{v}}_{t},{\overset{⌄}{x}}_{t}\right) \end{array}$$

(8d) $$\begin{array}{ll} & {\overset{⌄}{v}}_{t}={\overset{⌄}{v}}_{t}^{-}+\gamma \left(v_{t}-{\overset{⌄}{v}}_{t}^{-}\right) \end{array}$$

$$\begin{array}{ll} \; & {\overset{⌄}{v}}_{0}^{-}=v_{0},\;{\overset{⌄}{x}}_{0}=\xi \end{array}$$

where γ∈(0, 1) is a hyperparameter of the problem. Notice that setting γ = 0 turns (Equation 8) into a standard simulation-error system identification problem (Ljung, 1999), usually solved with GD and backpropagation through time; setting γ = 1 recovers the TF problem (Equation 3). Notice also that Equation 8d, which implements a convex combination of measurements and predictions, can also be interpreted as an *update* of the prediction ${\overset{⌄}{v}}_{t+1}^{-}$ using the data *v*_*t*+1_. The latter allows interpreting GTF for neuronal systems a method to learn a *filter* or *observer* (signal processing and control engineering terminologies, respectively) for the neuronal membrane voltage. This is how Abarbanel et al. (2009) interpreted their continuous-time version of the problem, which was applied to conductance-based models. The control theoretic interpretation allows one to explore further generalizations of teacher forcing involving, for instance, the Extended Kalman Filter, in which γ is made adaptive, see Burghi and Sepulchre (2024).

Because GTF in neuronal models exploits measured voltages but not measured internal states (the latter are not available), exploding gradients can in general still be observed during training. However, here we find another benefit of the contraction assumption (see *contraction theory* in Methods). If a neuronal data-driven model possesses a uniformly contracting internal dynamics, then there is a value of γ sufficiently close to 1 such that exploding gradients are precluded during gradient descent. But even if γ is chosen to avoid exploding gradients, one may still find that solving the problem (Equation 8) takes too much time, especially when the dataset length *N* is large. For that reason, one can *combine GTF with multiple shooting*. Doing so is trivial: the multiple shooting cost (Equation 7a) can be obtained by partitioning the GTF cost (Equation 8a) according to a desired shot length, and using the GTF constraints with γ = 0 within each shot. The combined GTF-multiple-shooting problem for γ>0 is obtained in the same way by keeping the GTF constraints (Equations 8b–8d) with γ>0.

**Figure 4:**
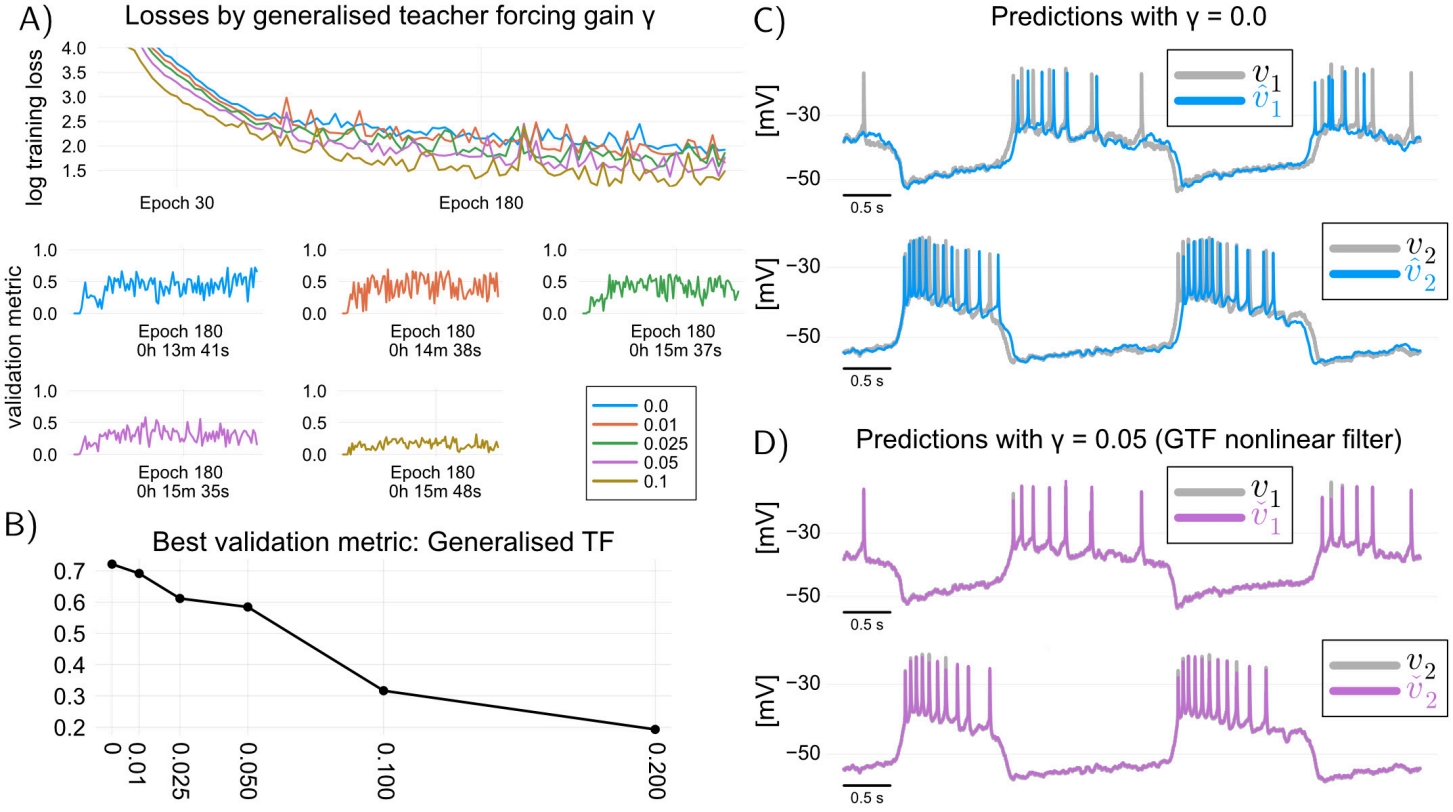
Generalized teacher forcing yields a data-driven filter or observer for the voltage dynamics. **(A)** learning curves when training the RMM (Equation 2) using GTF in combination with multiple shooting (shot length *s* = 20 samples). **(B)** There is no obvious benefit to using GTF if the purpose is to use data-driven models solely for simulation: trained model performance deteriorates for positive values of γ. **(C)** Simulation of trained model with γ = 0 (equivalent to MS). **(D)** Simulation of the filter equations for the model trained with GTF and γ = 0.05. GTF provides a nonlinear filter of the voltage dynamics which can be used to improve the quality of recordings, as well as to obtain better estimates of the internal states of the model.

An important question is whether combining GTF with multiple shooting yields better models than multiple shooting alone. Figure 4A illustrates typical training curves obtained with the combined GTF-multiple-shooting method, for different values of γ. It can be seen that increasing the value of γ>0 in general leads to poorer validation performance. This performance deterioration can be explained by the way we validate the models: in validation, models are not allowed to use the recorded voltages, and hence to obtain the validation curves of Figure 4A we have used predictions based on simulation of Equation 2, which does not contain the update step (Equation 8d) used to train a model with GTF. In other words, we have trained such models with γ>0, but validated them with γ = 0; a loss in performance is hence expected. This empirical observation seemingly questions the usefulness of GTF, at least in the context where it is used in conjunction with multiple shooting for speeding up training times.

In fact, GTF should not be contrasted with MS, but rather be viewed as method for training a discrete-time *nonlinear filter* of the membrane dynamics, which can be used to obtain improved measurements of the membrane potential, as well as improved estimates of its internal states. The filter is simply the Luenberger observer given by Equations 8b–8d. Figures 4C, D illustrate this point: we can see that the predictions ${\overset{⌄}{v}}_{t}$ of a simulation model (γ = 0.0) obtained with GTF can be drastically improved by generating predictions ${\overset{⌄}{v}}_{t}$ from the trained filter (γ = 0.05). The fact that very small values of γ result in good filter predictions suggests that predictive discrepancies in models trained with TF or multiple shooting (where γ = 0) can be at least in part attributed to *unmeasured input disturbances* that have not been taken into account when training those models; in fact, one interpretation of the correction term in an observer is that it provides an estimate of such disturbances (Sontag, 1998). Combining GTF with rapidly trainable data-driven models hence provides a means to filter out such disturbances, in real-time, if necessary.

#### 2.2.4 Training methods and model priors affect synaptic current predictions

**Figure 5:**
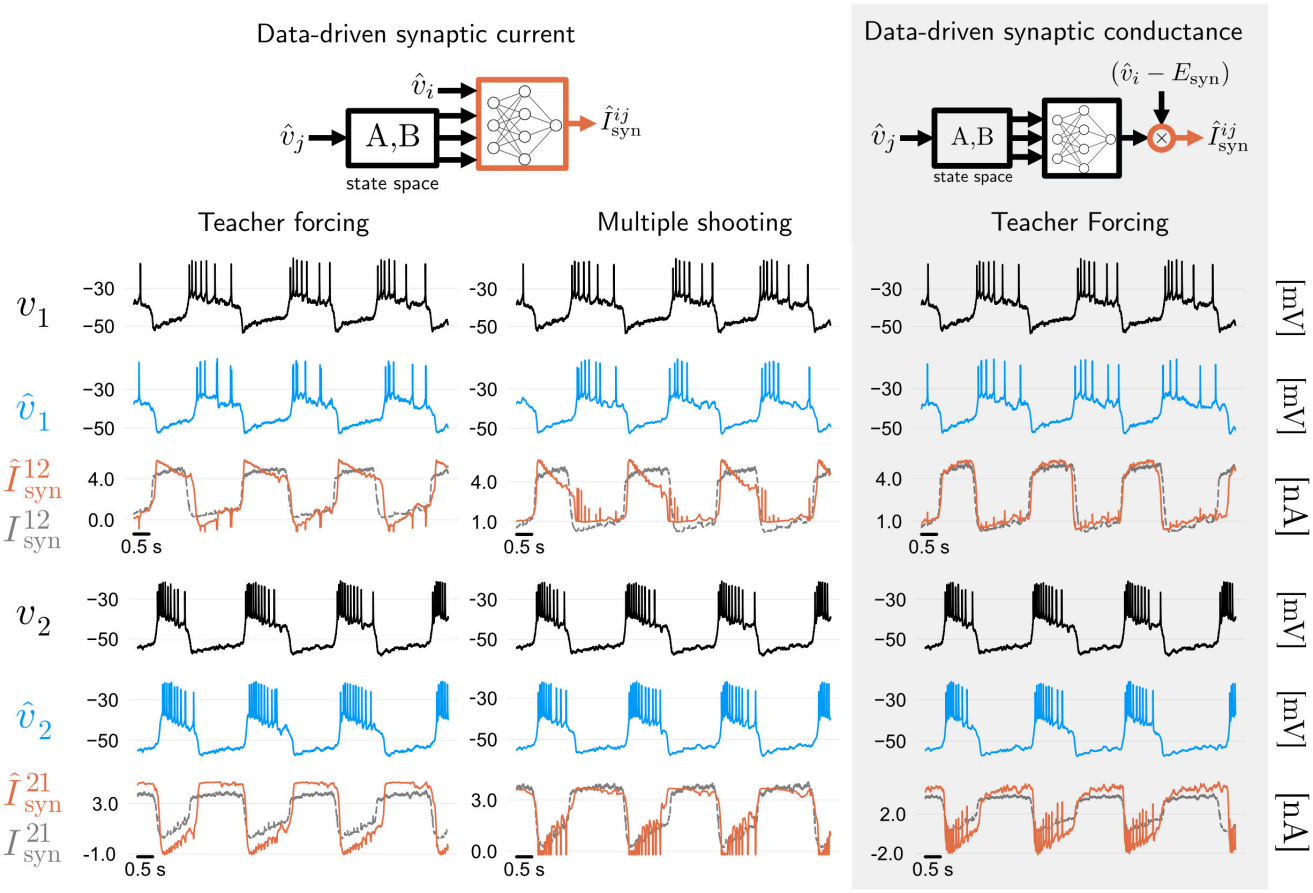
Synaptic current predictions in data-driven models are affected by training algorithms and model priors. Leftmost two panels: membrane voltage and synaptic current predictions of fully data-driven RMMs trained with feed-forward Teacher Forcing (TF) and Multiple Shooting (MS). Both methods yield a model with reasonable voltage prediction accuracy, with modified cosine similarities (see Methods) of around 0.73 and 0.8, respectively. Predictions of the synaptic currents are reasonably good in terms of magnitude and timing, but show a discrepancy with respect to the shape of the ground truth currents recorded during dynamic clamp (which were not used during training). This discrepancy can be attributed to degeneracy between intrinsic and synaptic data-driven currents. Rightmost panel: Adding biophysical priors to the data-driven architecture improves the shape of synaptic current predictions. Using TF, we trained an RMM (Burghi et al., 2025a) where synaptic conductances (rather than currents) are learned by artificial neural networks (see Methods). Biophysical priors given by the reversal potential and ANN constraints improves synaptic current predictions.

If intracellular voltage predictions of a neural circuit are all one cares about, then the takeaway from Figures 2–4 is that MS improves on TF, and GTF yields a nonlinear filter of the neuronal membrane, which can be used in real-time. However, how good are the predictions of the synaptic currents, which are not used during training? In the leftmost two panels of Figure 5, we show both voltage and synaptic current predictions of the best model trained with TF from Figure 2 (modified angular separation 0.73), and the best model trained with MS from Figure 3 (modified angular separation 0.8). While the model trained with MS predicts voltage better, it can be seen that synaptic current predictions of the TF and MS models suffer from the same problem: while those predictions are accurate in terms of magnitude and timing, the shape of the first synaptic current in both cases is inaccurate. This shows that the fully data-driven RMM from Equation 3.1 in Methods has a degenerate dynamics: some share of the ground truth synaptic current of the HCO is, in the RMM, supplied by the intrinsic currents. Given that the synaptic current is not used during training, this is a consequence of the fact that the RMM has very few priors—in fact, both its intrinsic and synaptic currents have the very same model structure.

We investigate whether synaptic current predictions can be improved by introducing biophysical priors in the data-driven model. This can be accomplished by employing *data-driven conductances* of RMMs (Burghi et al., 2025a). We replace the fully data-driven synaptic current (Equation 3.1 in Methods) by a biophysically informed version containing data-driven conductances (Equation 19 in Methods). After training the resulting RMM with teacher forcing, we obtain the predictions shown on the rightmost panel of Figure 5 (modified angular separation of around 0.75). It can be seen that including a biophysical prior on the data-driven model's synapse is enough to recover the shape of the first synapse to a high degree of accuracy, while the second synapse is predicted with a small error. This result shows that when it comes to predicting the internal currents of a neural circuit, one cannot be guided by the predictive accuracy of external (measured) signals alone.

### 2.3 Interpreting data-driven models of intracellular dynamics

A downside of using fully data-driven models of the form (Equation 1) is the fact that it is not trivial to interpret the model's dynamics in biophysical terms. We show that data-driven models can still be interpreted in quasi-biophysical terms by means of a *frequency-dependent conductance*. This is a generalization of the familiar *input conductance* used by eletrophysiologists to study neuronal excitability (Mauro et al., 1970; Franci et al., 2019). Frequency-dependent conductances uncover excitable properties of the membrane at different timescales, serving a purpose similar to that of the early and late IV curves originally introduced by Hodgkin and Huxley (1952). This section discusses such conductances by assuming that Equation 1 is the model of a single neuron (*n* = 1).

#### 2.3.1 Frequency-dependent conductances

We derive frequency-dependent conductances of a data-driven model satisfying the contraction assumption (Methods). This assumption ensures that the system (Equation 1) has a (steady-state) IV curve given by


(9a) $$\begin{array}{l} u_{\infty }\left(v\right)=h\left(v,x_{\infty }\left(v\right)\right) \end{array}$$

where *x*_∞_(*v*) is the unique equilibrium state of Equation 1b as a function of *v*, that is, it satisfies


(9b) $$\begin{array}{l} x_{\infty }\left(v\right)=f\left(v,x_{\infty }\left(v\right)\right) \end{array}$$

for any *v*. The fact that this unique equilibrium exists is a consequence of the contraction of the internal dynamics (Equation 1b), which for constant *v* becomes an autonomous system (Lohmiller and Slotine, 1998, Section 3). The derivative of the IV curve (Equation 9a) is the (steady-state) *condutance-voltage (GV) curve*. It is given by


(10) $$\begin{array}{l} G_{\infty }\left(v\right)={\left[\frac{\partial h}{\partial v}+\frac{\partial h}{\partial x}\frac{\partial x_{\infty }}{\partial v}\right]}_{v=v,x=x_{\infty }\left(v\right)} \end{array}$$

which follows from the chain rule. The *admittance-voltage (YV)* curve is an extension of the GV curve into the frequency domain. For discrete-time circuits, it is given by


(11) $$\begin{array}{l} Y\left(v,\omega \right)={\left[\frac{\partial h}{\partial v}+\frac{\partial h}{\partial x}{\left(e^{j\omega \delta }I-\frac{\partial f}{\partial x}\right)}^{-1}\frac{\partial f}{\partial v}\right]}_{v=v,x=x_{\infty }\left(v\right)} \end{array}$$

where *I* is the identity matrix. For an explanation of Equation 11, see *admittance-voltage curve* in Methods. The YV curve is complex-valued, and hence can be decomposed into


(12) $$\begin{array}{l} G\left(v,\omega \right)=\text{Re}\left[Y\left(v,\omega \right)\right],\;B\left(v,\omega \right)=\text{Im}\left[Y\left(v,\omega \right)\right] \end{array}$$

which are the *frequency-dependent conductance* and susceptance, respectively. In Methods, we show that


(13) $$\begin{array}{l} G\left(v,0\right)=G_{\infty }\left(v\right) \end{array}$$

which formally demonstrates that *G*(*v*, ω) extends the GV curve into the frequency domain.

#### 2.3.2 Voltage-frequency regions of excitability

Electrophysiologists have long inferred neuronal excitability from experimentally recorded steady-state IV curves exhibiting regions of negative slope—negative conductances. These are regions where, locally, ionic currents tend to collectively destabilize the membrane dynamics. In a data-driven model, this occurs at voltages where *G*_∞_(*v*) < 0. It is however well-known that negative steady-state conductances are by no means necessary for excitable behavior. Frequency-dependent conductances generalize the previous statements by taking membrane dynamics into account. Formally, we can infer that a model is (locally) excitable on a voltage range *V* if we can find a range of frequencies Ω where


(14) $$\begin{array}{l} G\left(v,\omega \right)<0 \end{array}$$

for all (*v*, ω)∈*V*×Ω. If 0∈Ω, then Equation 14 includes the steady-state case *G*_∞_(*v*) < 0. If Ω contains positive frequencies, then we can say that the model presents *frequency preference*: the neuron responds excitably to frequencies where Equation 14 holds (see Izhikevich, 2007, p. 232 for a dynamical systems theory point of view). Control theory provides an interesting interpretation of the local excitability condition (Equation 14). A stable linear system is said to be *passive* if their complex-valued frequency response is confined to the closed right half of the complex plane (Goodwin and Sin, 1984). Since the admittance *Y*(*v*, ω) represents the frequency response of the (linearized) total membrane current, we can interpret Equation 14 as a passivity-breaking condition on the total membrane current dynamics. In other words, Equation 14 indicates the presence of *active* currents acting at timescales given by 2π/ω.

**Figure 6:**
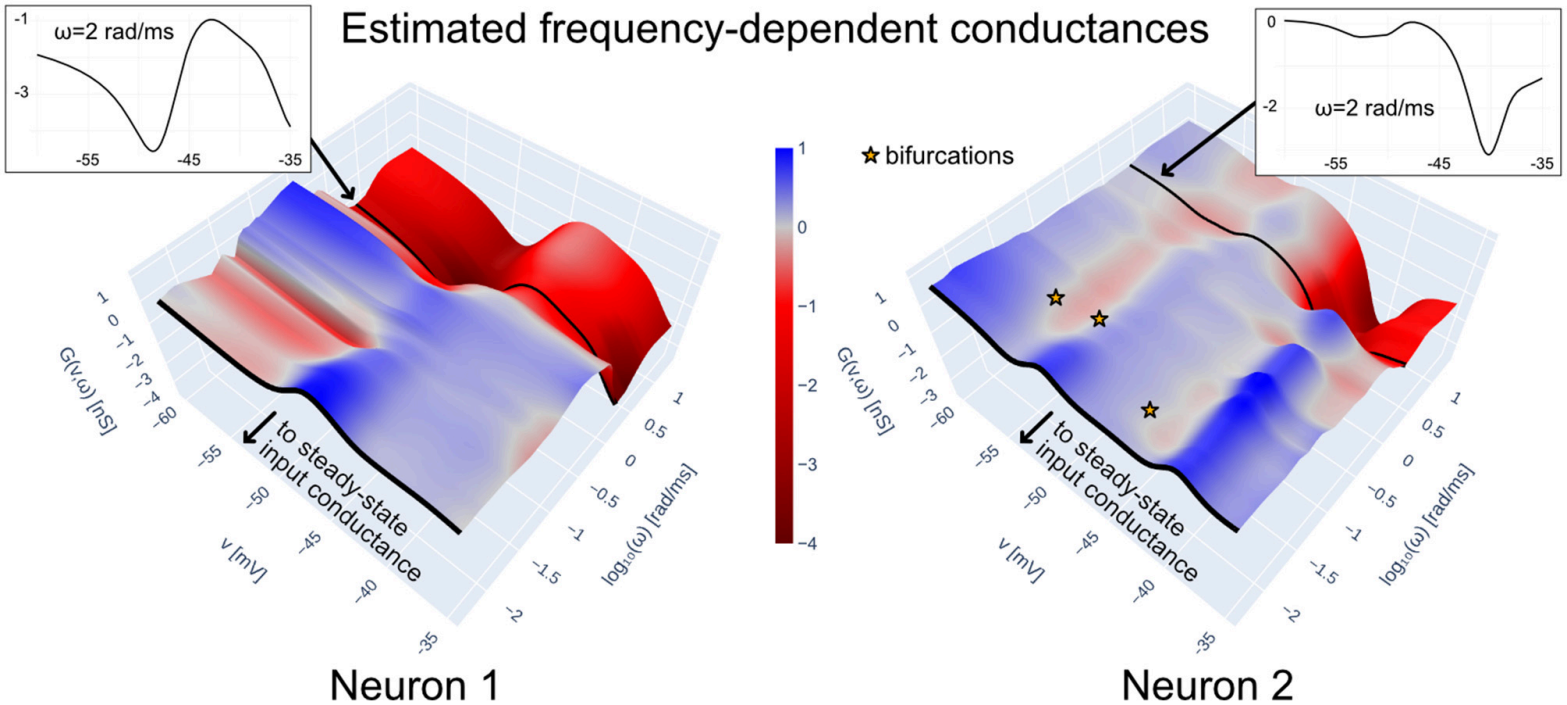
Frequency-dependent conductances of Î_int_+Î_leak_ for each of the two data-driven neuron models in the HCO of Figure 1C, trained with teacher forcing; synaptic currents are implemented with biophysical priors according to Methods (see also Figure 5). Frequency-dependent conductances generalize the steady-state input conductance by taking into account the frequency dependence of the linearized dynamics of Î_int_+Î_leak_ as a function of voltage. They allow interpreting a data-driven neuron in terms of regions of excitability, where *G*(*v*, ω) < 0. The frequency dependent conductances above are consistent with a burst-excitable model: both models possess negative conductances at low voltages and low frequencies, with a peak of positive conductances at higher voltages and low frequencies. Slow negative conductances are consistent with the *I*_h_ currents in each neuron, which were included in the preparation via dynamic clamp. Negative conductances at higher frequencies account for the existence of biological sodium channels in both neurons.

To illustrate the above points, Figure 6 shows the frequency-dependent conductance of the total intrinsic current of each of the neurons in the HCO model constructed with data-driven conductance-based synapses (see Equations 3.1 and 19 in Methods; the trained model corresponds to the rightmost pane of Figure 5). Intrinsic admittances of each neuron are computed with Equations 11–12 using $h={Î}_{\text{int}}^{i}+{Î}_{\text{leak}}^{i}$ for *i* = 1, 2. The frequency-dependent conductances in Figure 6 are in accordance to the excitability features of the biological neurons in the HCO. Both neuron models are locally excitable (red regions) at low voltages and low frequencies, a feature we can attribute to the *I*_h_ (hyperpolarization-induced) present in the preparation. Furthermore, the conductance of Neuron 2 near −40 mV is considerably more negative than that of Neuron 1; this agrees with Neuron 1's sharp decrease in intra-burst excitability around that voltage (cf. Figure 4).

#### 2.3.3 Ultrasensitivity and bifurcations

In neurons with non-monotonic IV curves, containing negative steady-state conductances, one finds that the onset of spiking or bursting occurs at the boundary of the voltage interval where *G*_∞_(*v*) < 0. Frequency-dependent conductances enable a generalization of this statement. Formally, any voltage $\bar{v}$ where


(15) $$\begin{array}{l} G_{\infty }\left(\bar{v}\right)=0\text{ and }G_{\infty }^{\prime }\left(\bar{v}\right)\neq 0 \end{array}$$

lies at a *saddle-node* (also called *fold*) bifurcation point (Izhikevich, 2007). In such cases, increasing the applied current of an initially quiescent model past $u_{\infty }\left(\bar{v}\right)$ pushes the voltage past the threshold $\bar{v}$ toward spiking or bursting. For excitable models with a monotonic steady-state IV curve, Equation 15 cannot be satisfied, and a more general condition is required. In Methods, we show that the condition


(16) $$\begin{array}{l} C\left(\cos\left(\bar{\omega }\delta \right)-1\right)+\delta G\left(\bar{v},\bar{\omega }\right)=0 \end{array}$$

is a necessary condition for a saddle-node or Neimark-Sacker (the discrete-time version of the Hopf) bifurcation when $\bar{\omega }=0$ or $\bar{\omega }>0$, respectively, generalizing the condition (Equation 15) by means of the relation (Equation 13). The candidate bifurcation point $\left(\bar{v},\bar{\omega }\right)$ can be found by solving Equation 30 in Methods. Critically, for small sampling time δ, Equation 16 occurs near the boundary of the regions of negative conductance given by Equation 14.^1^ Borrowing terminology from Franci et al. (2019), in this region of the frequency-voltage space, the model can be *ultra-sensitive*.

Returning to Figure 6, we have plotted the candidate bifurcation points of Neuron 2 in the HCO model; recalling that the admittance is computed with $h={Î}_{\text{int}}^{2}+{Î}_{\text{leak}}^{2}$, this analysis treats the synaptic current from Neuron 1 as an external input. It can be seen that those points occur close to where the conductance flips sign. Furthermore, we can see that a bifurcation is predicted near −54 mV, which agrees well with the onset of the Neuron 2 bursts seen in Figure 5.

## 3 Methods

### 3.1 Modeling details

The RMM (Equation 2) used to model the HCO circuit of Figure 1 is given by


$$\begin{array}{l} c_{i}\frac{{\hat{v}}_{i,t+1}-{\hat{v}}_{i,t}}{\delta }=-\underset{{Î}_{\text{int},t}^{i}}{\underset{︸}{\mathrm{mlp}\left({\hat{v}}_{i,t},w_{i,t};\theta_{i}^{\text{int}}\right)}}-\underset{{Î}_{\text{syn},t}^{ij}}{\underset{︸}{\mathrm{mlp}\left({\hat{v}}_{i,t},z_{i,t};\theta_{i}^{\text{syn}}\right)}}\\ \;-\underset{{Î}_{\text{leak},t}^{i}}{\underset{︸}{\theta_{i}^{\text{leak},1}{\hat{v}}_{i,t}+\theta_{i}^{\text{leak},2}}}+u_{i,t}\\ \;w_{i,t+1}=A_{i}^{\text{int}}w_{i,t}+B_{i}^{\text{int}}{\hat{v}}_{i,t}\\ \;z_{i,t+1}=A_{i}^{\text{syn}}z_{i,t}+B_{i}^{\text{syn}}{\hat{v}}_{j,t} \end{array}$$

with δ = 0.1 the sampling period of the data. Here, $\text{mlp}\left({\hat{v}}_{i,t},w_{i,t};\theta_{i}^{\text{int}}\right)$ is a multi-layer perceptron (MLP) representing the sum total of intrinsic currents flowing through neuron i, while $\text{mlp}\left({\hat{v}}_{i,t},z_{i,t};\theta_{i}^{\text{syn}}\right)$ represents the total synaptic current flowing from neuron j to neuron i. The MLPs are given by the chained composition of matrix multiplications and bias additions with sigmoidal activation functions, which are in turn composed with an input normalization layer. Mathematically,


(17) $$\begin{array}{l} \text{mlp}\left(v,w;\theta \right):=W_{L+1}{○}_{\ell =1}^{L}\sigma_{L}\left(W_{\ell }\cdot +b_{\ell }\right){}^{\circ}q\left(v,w\right) \end{array}$$

where: the circle operator denotes function composition, *W*_ℓ_ and *b*_ℓ_ are learnable layer weights and biases, constituting the parameter vector θ; σ_ℓ_ = tanh for all ℓ are activation functions applied elementwise to their inputs; and *q*(*v, w*) is an affine normalization function mapping the minimum and the maximum values of *v* and *w*_*i*_ of the training dataset to −1 and +1, respectively (the *w* states of the training dataset are obtained by warming up; see *training details*). The use of an MLP (as opposed to a single-layer perceptron) can be justified on the basis that, since the internal dynamics in Equation 3.1 is linear, more complexity in the voltage dynamics may be desirable (empirically, few layers are required for satisfactory results). In this paper, the intrinsic current MLP has *L*^int^ = 3 layers with 20 activation functions in each layer, and the synaptic current MLP has *L*^syn^ = 3 layers with 10 activation functions in each layer. The parameters $\theta^{\text{leak},1}={\left({\tilde{\theta }}^{\text{leak},1}\right)}_{+}$ and θ^leak, 2^ are used to represent the leak current, with the former being constrained to be positive by the softplus function (·)_+_ so that it implements a positive leak conductance. MLP weights were initialized with the Nguyen-Widrow initialization heuristic (Nguyen and Widrow, 1990), with biases initialized spread over the interval -1 to 1.

Following Burghi et al. (2025a), the linear systems in the internal dynamics of Equation 3.1 are constructed so as to have orthogonal impulse responses (Heuberger et al., 2005), which is done to produce a rich set of state trajectories following a voltage spike. To construct the discrete-time system matrices *A* and *B*, we consider prior knowledge about the continuous time constants relevant to neural dynamics. Starting from a set of continuous time constants {_τ_*k*_}*k* = 1, …, *n*_, we first discretize the τ_*k*_ with the stability-preserving transformation


$$\lambda_{k}=\exp\left(-\delta /\tau_{k}\right)$$

(recall δ is the sampling period). We then set *A*: = *A*_*n*_ and *B*: = *B*_*n*_, with *A*_*n*_ and *B*_*n*_ being computed according to


(18) $$\begin{array}{l} A_{1}=\lambda_{1},\;B_{1}=\sqrt{1-\lambda_{1}^{2}},\;\Gamma_{1}=B_{1},\;\Delta_{1}=-\lambda_{1}\\ \;A_{i}=\left[\begin{array}{cc} A_{i-1} & 0\\ \sqrt{1-\lambda_{i}^{2}}\Gamma_{i-1} & \lambda_{i} \end{array}\right],\;B_{i}=\left[\begin{array}{c} B_{i-1}\\ \sqrt{1-\lambda_{i}^{2}}\Delta_{i-1} \end{array}\right]\\ \Gamma_{i}=\left[\begin{array}{cc} -\lambda_{i}\Gamma_{i-1} & \sqrt{1-\lambda_{i}^{2}} \end{array}\right],\;\Delta_{i}=-\lambda_{i}\Delta_{i-1} \end{array}$$

for *i* = 2, …, *n*. The resulting state space system responds to an impulse function with orthogonal state trajectories, see (Heuberger et al., 2005, Chapter 2) for details and the SI appendix of Burghi et al. (2025a) for an illustration. To construct *A*^int^ and *A*^syn^, we define the two time constant subsets


$$\begin{array}{l} \tau^{\text{fast}+\text{slow}}={\left\{0.2k\right\}}_{k=1,\ldots ,8}\cup {\left\{2.0k\right\}}_{k=1,\ldots ,8}\cup {\left\{10+10k\right\}}_{k=1,\ldots ,8}\\ \;\tau^{\text{ultraslow}}={\left\{200k-100\right\}}_{k=1,\ldots ,8} \end{array}$$

which were chosen by sampling the range of the time constant functions found in the gating kinetics of conductance-based models of neurons from the STG (Liu et al., 1998) [see the SI appendix of Burghi et al. (2025a) for plots of such functions]. The matrix *A*^int^ is constructed as in Equation 18 using τ^fast+slow^∪τ^ultraslow^, while *A*^syn^ is constructed with τ^fast+slow^ only. This results in a total of 96 internal states in the full data-driven HCO model. In this paper the *A* and *B* matrices are kept fixed to illustrate how feed-forward teacher forcing (which precludes learning those matrices) can be related to MS and GTF. While it is in principle possible to learn the components of *A* and *B* during MS and GTF, we do not pursue this in this paper.

#### 3.1.1 Synaptic current with data-driven conductances

To obtain an HCO model with biophysically informed data-driven synapses (Burghi et al., 2025a), we replace the synaptic current model in Equation 3.1 by


(19) $$\begin{array}{l} \;{Î}_{\text{syn},\text{t}}^{ij}={ḡ}^{ij}\sigma_{+}\left(h_{+}\left(z_{i,t}\right);\theta_{i}^{\text{syn}}\right)\left({\hat{v}}_{i,t}-E^{\text{syn}}\right)\\ z_{i,t+1}=\text{diag}\left(\lambda \left[A_{i}^{\text{syn}}\right]\right)z_{i,t}+\left(1-\lambda \left[A_{i}^{\text{syn}}\right]\right){\hat{v}}_{j,t} \end{array}$$

where we have used λ[·] to represent the vector of eigenvalues of a matrix, and **1** to represent the all-ones vector. In Equation 19, $\sigma_{+}\left(\cdot ;\theta_{i}^{\text{syn}}\right)$ is a single-layer perceptron whose weight matrices are constrained to be elementwise positive, and *h*_+_(·) is a fixed min-max-type normalization layer constructed so that, at random parameter initialization, the output of σ_+_ is approximately 0 at −60.0 mV, and approximately 1 at −40.0; this is done to mimic the activation range of the synapse used in the dynamic clamp experiment [see *constructing data-driven conductances* in the Methods section of (Burghi et al. 2025a) for details]. The activation ranges above are not fine-tuned, so that the model can be said to be parsimonious one. The scalar ḡ is a learnable maximal conductance parameter, and *E*^syn^ is a fixed Nersnt potential with value set to that of the synapse implemented in the HCO with dynamic clamp.

### 3.2 Contraction theory

We identify a tractable mathematical condition on the generic model (Equation 1) that guarantees the well-posedness of training and interpretability methods:

**Exponential contraction condition:** In the generic data-driven model of Equation 1, the internal dynamics states *x*_*t*_ in Equation 1b are *exponentially contracting* (Lohmiller and Slotine, 1998), uniformly in the voltage variable *v*_*t*_ and the parameters η.

Exponential contraction implies that for any given trajectory of *v*_*t*_, the distance between two trajectories of *x*_*t*_ starting at different points of the state space decreases exponentially fast as *t* → ∞ (Theorem 2 of Lohmiller and Slotine, 1998). A key technical feature of exponential contraction is that it can be guaranteed to hold via a sufficient condition involving a Lyapunov-type linear matrix inequality, which can be verified efficiently. Mathematically, contraction of the internal states is guaranteed if one can find a constant positive definite matrix^2^ *P* and a positive constant 0 < α < 1 such that the Jacobian of the internal dynamics vector field satisfies the linear matrix inequality (LMI) given by


(20) $$\begin{array}{l} \frac{\partial f_{\eta }}{\partial x}{\left(v,x\right)}^{⊺}P\frac{\partial f_{\eta }}{\partial x}\left(v,x\right)≼\alpha P \end{array}$$

for all *x* and *v* (Lohmiller and Slotine, 1998). Intuitively, contraction is a strong stability property which makes a nonlinear system behave in many ways similarly to a stable linear one; for a tutorial, see Jouffroy and Fossen (2010). For data-driven models, one can guarantee the that the internal states *x*_*t*_ contract throughout the learning procedure and by constraining the internal dynamics (Equation 1b) so that the matrix inequality holds uniformly in the parameters η.

When the internal dynamics of a data-driven model is linear, as is the case of Equation 2, then it is well known (Goodwin and Sin, 1984) that the LMI condition (Equation 20) becomes equivalent to the simpler condition below:

**Exponential contraction condition (simple case):** In the Recurrent Mechanistic Model of (Equation 2), we have

(21) $$\begin{array}{l} |\lambda_{i}\left[A\right]|<1,\;i=1,\ldots ,\text{dim}\left(x_{t}\right) \end{array}$$

where λ_*i*_[*A*] denotes the *i*^th^ eigenvalue of *A*. In other words, the dynamics of *x*_*t*_ with *v*_*t*_ seen as an input is asymptotically stable.

The stability condition above is simple to enforce in linear systems, and is guaranteed by the constructive procedure used to obtain *A* described in the previous section.

#### 3.2.1 Contraction and training methods

While weaker conditions with Lyapunov exponents can also achieve the effect of trajectory convergence seen in contracting systems, what makes dealing with contraction matrix inequalities such as Equation 20 attractive is the fact that it can be used to show that training with TF, MS, and GTF is tractable.

When the contracting internal dynamics assumption above is satisfied, then it is possible to guarantee that exploding gradients will not be observed in teacher forcing (TF) nor in generalized teacher forcing (GTF), regardless of the time horizon of the dataset. In TF, this can be shown by applying the chain rule to the loss gradient ∇L, and then using the LMI (Equation 20) to ensure that the gradient does not grow unbounded as the number of datapoints grows as *N* → ∞. For instance, when computing $\nabla_{\eta }L$, one obtains a term $\nabla_{\eta }{\hat{x}}_{N-1}\nabla_{x}h_{\theta }\left({\bar{v}}_{N-1},{\hat{x}}_{N-1}\right)\left({\bar{v}}_{N}-{\hat{v}}_{N}\right)$ that could potentially grow unbounded. This is because $\nabla_{\eta }{\hat{x}}_{N-1}$ is given by the recurrence relation


(22) $$\begin{array}{l} \nabla_{\eta }{\hat{x}}_{t}=\nabla_{\eta }{\hat{x}}_{t-1}\nabla_{x}f_{\eta }\left({\bar{v}}_{t-1},{\hat{x}}_{t-1}\right)+\nabla_{\eta }f_{\eta }\left({\bar{v}}_{t-1},{\hat{x}}_{t-1}\right) \end{array}$$

which is a (potentially unstable) time-varying linear system. But the contraction LMI (Equation 20) ensures that this dynamical system has bounded states; this can be shown by taking $\left(\nabla_{\eta }{\hat{x}}_{t}^{⊺}\right)P\left(\nabla_{\eta }{\hat{x}}_{t}\right)$ as a Lyapunov function for Equation 22 (see Khalil, 2002, Chapter 4 for an introduction to bounded-input-bounded-state stability analysis). In GTF, the situation becomes complicated by the interaction between ${\hat{v}}_{t}$ and ${\hat{x}}_{t}$. But using contraction analysis, it can be claimed that under the contracting internal states assumption, taking γ close enough to 1 ensures that the dynamical system given by Equation 8b–8d is also contracting, and hence gradients cannot explode, similarly to the TF case. The proof of this claim is a simple discrete-time reformulation of Proposition 2 in Burghi et al. (2021).

### 3.3 Training details

All models in this paper were programmed in Julia using the Flux.jl package (Innes, 2018) for implementing MLPs as well as the Adam (Kingma and Ba, 2017) gradient descent routine. The code used to implement this model can be found on github.com/thiagoburghi/RMM/tree/HCO. The Adam moment parameters used in this paper are given by β_1_ = 0.9 and β_2_ = 0.999 in all cases. For models trained with TF in Figures 2, 5, and 6, we used a step size of η = 0.001. For models trained with MS in Figures 3 and 5, we used a scheduler for the step size, starting at η = 0.01 and changing to η = 0.005 at 50 epochs and η = 0.0025 at 100 epochs (this was done to rapidly decrease the large initial error in shot predictions). For models trained with GTF in Figure 4, we used a fixed step size o η = 0.01. The *D*-norm in Equation 7a is a weighted 2-norm; we have used a diagonal weight matrix *D* whose nonzero entries are given by the inverse of the DC gain (*I*−*A*)^−1^*B*. MS and GTF were implemented by partitioning the dataset into 48 mini-batches, and applying the methods as described in Equations 7 and 8 within each mini-batch before taking a gradient step. Each epoch in Figures 2–4 contains one gradient update for each mini-batch; mini-batches in MS and GTF were shuffled between each epoch (notice this is different from shuffling in TF, which also shuffles samples within mini-batches). All models were trained from randomly initialized MLP parameters.

#### 3.3.1 Warming up initial conditions

Warming up the internal states' initial conditions is one of the most important steps in training data-driven models using the methods described in this paper. Consider a generic model in the form (Equation 1) satisfying the exponential contraction condition (Equation 20). Given the voltage data *v*_*t*_ and an initial parameter vector η_0_, let


$${\bar{x}}_{t+1}^{0}=f_{\eta^{0}}\left(v_{t},{\bar{x}}_{t}^{0}\right),\;t=-M,-\left(M-1\right),\ldots ,-1,0,1,\ldots ,N-1$$

where *v*_−*M*_, *v*_−(*M*−1)_, …, *v*_−1_ is an initial portion of the data that is discarded during training, and *v*_0_ and *v*_*N*−1_ are the first and last data points used during training, respectively. Under the assumption of contraction, the value of ${\bar{x}}^{-M}$ does not matter, as it is forgotten along with the internal dynamics' transients. The warmed up states can be used as follows: in the teacher forcing problems (Equations 3a and 5), one simply chooses $\xi^{0}={\bar{x}}_{0}^{0}$, and in the multiple shooting problem (Equation 7a) (and when combining it with GTF), one takes $\xi^{\left(n\right)}={\bar{x}}_{ns}^{0}$ for *n* = 0, 1, …, *N*_*s*_.

### 3.4 Applied current

To create the HCO, the two GM cells were virtually coupled together with dynamic clamp (Sharp et al., 1993). This was achieved using a custom RTXI module (Patel et al., 2017) to inject voltage-dependent currents into each cell. The total applied current injected in the *i*^th^ HCO neuron is given by


(23) $$\begin{array}{l} I_{\text{app},t}^{i}=\underset{\text{dynamic clamp}}{\underset{︸}{I_{\text{h},t}^{i}+I_{\text{syn},t}^{ij}}}+u_{i,t} \end{array}$$

where (*i, j*)∈{(1, 2), (2, 1)}. In Equation 23, the current components $I_{\text{h},t}^{i}$ and $I_{\text{syn},t}^{ij}$ are a virtual hyperpolarization-induced current and a synaptic current defined with conductance-based models, while the current *u*_*i, t*_ is a stochastic current used to perturb the HCO dynamics and train the data-driven model. Each individual current component in Equation 23 was saved separately by RTXI while measuring the responses of the HCO.

#### 3.4.1 Noisy injected current

The current vector *u*_*t*_ in Equations 1, 2, 3, 7 and 8 represents a measured injected current used to excite the dynamics of a neural circuit and facilitate the training of a data-driven model. In the context of our HCO experiment, *u*_*t*_ = (*u*_1, *t*_, *u*_2, *t*_) with *u*_*i, t*_ being defined as a stochastic component of the applied current in Equation 23. Each *u*_*i, t*_ is given by a discretized Ornstein-Uhlenbeck (OU) process with nonzero mean,


(24) $$\begin{array}{l} \begin{array}{c} u_{i,t+1}=u_{i,t}+\delta a\left(\mu_{i}-u_{i,t}\right)+b\sqrt{\delta }\varepsilon_{i,t} \end{array} \end{array}$$

where δ = 0.1 ms is the sampling period, μ_*i*_ is the process mean, *a* and *b* are process parameters determining the process rate and variance, respectively, and ε_*i, t*_ are i.i.d. normally distributed random variables with zero mean and unity standard deviation. All the models in this paper were trained and validated with the same dataset, which consisted in two trials of around two minutes each, obtained by changing the parameters *a* and *b*. We fixed the means μ_*i*_ to values where a rhythm could be observed (see also *dynamic clamp* below), and then recorded two minutes of data with the following combinations of OU process parameters: (*a, b*)= (0.04, 0.1), (0.02, 0.025). Training was performed using the first 75% percent of the dataset (around 165 seconds), while validation was performed using the last 25% of one of the trials (around 27 seconds).

#### 3.4.2 Dynamic clamp currents

Similarly to Morozova et al. (2022), we used the synapse model


(25) $$\begin{array}{l} I_{\text{syn},t}^{ij}=-{ḡ}_{\text{syn}}^{i}z_{i,t}\left(v_{i,t}-E_{\text{syn}}^{i}\right)\\ z_{i,t+1}=z_{i,t}+\frac{\delta }{50\left(1.1-\sigma_{\text{syn}}^{i}\left(v_{j,t}\right)\right)}\left(-z_{i,t}+\sigma_{\text{syn}}^{i}\left(v_{j,t}\right)\right) \end{array}$$

where δ is the sampling period, and $\sigma_{\text{syn}}^{i}\left(v_{j,t}\right)={\left(1+\exp\left(-\left(v_{j,t}-\nu_{\text{syn}}^{i}\right)/2\right)\right)}^{-1}$ is the logistic synaptic activation function. Parameters of each of the two synapses can be found in Table 1. The hyperpolarization-induced current was given by


(26) $$\begin{array}{l} \;I_{\text{h},t}^{i}=-{ḡ}_{\text{h}}^{i}w_{i,t}\left(v_{i,t}-E_{\text{h}}^{i}\right)\\ w_{i,t+1}=w_{i,t}+\frac{\delta }{{\bar{\tau }}_{i}\tau_{\text{h}}\left(v_{i,t}\right)+0.1}\left(-w_{i,t}+\sigma_{\text{h}}^{i}\left(v_{i,t}\right)\right) \end{array}$$

with $\tau_{\text{h}}(v_{i,t})=(1+\exp{\left(-(v_{i,t}+110)/13\right)}^{-1}$ and $\sigma_{\text{h}}^{i}\left(v_{i,t}\right)=\left(1+\exp\left(-\left(v_{i,t}-\nu_{\text{h}}^{i}\right)/7\right)\right)$ both given by logistic nonlinearities. The specific parameters of each hyperpolarization-induced current are found in Table 1. Synaptic and H-current parameters were chosen starting from the values indicated in Morozova et al. (2022), and then varying the parameters shown in Table 1 until a HCO rhythm was observed under dynamic clamp.

**Table 1:** Parameters used to create the HCO with dynamic clamp.

***i***	** ${ḡ}_{\text{syn}}^{i}$ **	** $E_{\text{syn}}^{i}$ **	** $\nu_{\text{syn}}^{i}$ **	** ${ḡ}_{\text{h}}^{i}$ **	** $E_{\text{h}}^{i}$ **	** $\nu_{\text{h}}^{i}$ **	** ${\bar{\tau }}_{i}$ **
1	105	-80	-50 mV	225	-10	-45 mV	1,000 ms
2	150	-80	-44 mV	120	-10	-45 mV	1,500 ms

#### 3.4.3 Validation

In Figures 2–4, the *training loss* corresponds to the loss function of the method being used to train the model, that is, Equations 6, 7a or 8a. The *validation metric* is a modified angular separation metric used to assess the predictive power of the models when they are used for predicting the voltage time series, which is done by simulating (Equation 2). The angular separation between two smoothed spike trains is a traditional metric for comparing the similarity between two spike trains (Gerstner et al., 2014), and is given by 〈*y*, ŷ〉/(||*y*||||ŷ||) where *y* and ŷ are vector-valued spike train sequences, 〈·, ·〉 is the inner product, and ||·|| is the norm of a vector. In this work we modified this metric according to


$$\text{modified angular separation}=\frac{\langle y,ŷ\rangle }{\max\left\{||y{||}^{2},||ŷ{||}^{2}\right\}}$$

so as to take magnitude (as well as shape) of the smoothed spike trains into account. The modified metric was empirically determined to work better for bursting signals. Here, *y* = {*y*_0_, …, *y*_*N*_} and ŷ = {ŷ_0_, …, ŷ_*N*_} are smoothed spike train time series obtained as follows: (i) both voltage time series *v* and $\hat{v}$ are bandpass-filtered forwards and backwards in time by a 3^th^ order Butterworth filter with cutoff frequencies at 1/50 and 1/2 kHz to remove the slow bursting wave (forwards-and-backwards filtering is used to avoid phase distortion) (ii) the result is thresholded using a relu nonlinearity with threshold set at 5 mV to extract the spikes from the resulting high-frequency spiking signal iii) the maxima of thresholded spikes is used to define a spike train of impulses (maxima within 5 samples counted as a single spike) iv) the resulting spike train is convolved with a Laplace kernel given by $\exp\left(-|t|/500\right)/\underset{t}{\sum }|\exp\left(-|t|/500\right)|$. Notice that the modified angular separation always lies between 0 and 1, with 0 corresponding to completely disjoint smoothed spike trains, and 1 corresponding to a perfect prediction.

### 3.5 Admittance-voltage curve

The YV curve *Y*(*v*, ω) in Equation 11 describes how a small sinusoidal signal of frequency ω around a constant set-point *v* is amplified by the total intrinsic current of the neuron (the sum of the leak and ionic currents, and, in a neuronal circuit, of the synaptic currents). Mathematically, this signal can be represented as


$${ṽ}_{t}=\varepsilon \sin\left(\omega \delta t\right)+v,\;t=0,1,2,\ldots$$

with constant *v* and small ε>0 (recall δ is the sampling period of the measurements). The total intrinsic current of a neuron (or circuit) is the dynamical system


(27) $$\begin{array}{l} x_{t+1}=f_{\eta }\left(v_{t},x_{t}\right)\\ \;y_{t}=h_{\theta }\left(v_{t},x_{t}\right) \end{array}$$

with input given by *v*_*t*_ and output (the current) given by *y*_*t*_. The YV curve (Equation 11) is the *transfer function* of the *linearized* system above (Aström and Murray, 2008). It can be used to determine that the approximate response of the system to the sinusoidal signal, according to


(28) $$\begin{array}{l} y_{t}=\varepsilon |Y\left(v,\omega \right)|\sin\left(\omega \delta t+∠Y\left(v,\omega \right)\right)+u_{\infty }\left(v\right),\;t=0,1,2,\ldots \end{array}$$

where |*Y*(*v*, ω)| is the *gain* and ∠*Y*(*v*, ω) the *phase* of the system's transfer function.

To derive the extension of conductances into the frequency domain, we prove the following statement:

**Proposition:** Under the contraction assumption, we have


(29) $$\begin{array}{l} Y\left(v,0\right)=G_{\infty }\left(v\right) \end{array}$$

for all *v*. Furthermore, if there exist $\bar{\omega }\geq 0$ and $\bar{v}\in 0$ such that


(30) $$\begin{array}{l} C\left(e^{j\bar{\omega }\delta }-1\right)+\delta Y\left(\bar{v},\bar{\omega }\right)=0 \end{array}$$

then $\bar{v}$ is a candidate fold bifurcation point ($\bar{\omega }=0$) or a candidate Neimark-Sacker bifurcation point ($\bar{\omega }>0$).

*Proof of the proposition:* The contraction assumption ensures that, for constant *v*, the nonlinear system (Equation 27) has a unique equilibrium point *x*_∞_(*v*) which is asymptically stable (Theorem 2 of Lohmiller and Slotine, 1998). This equilibrium point is given implicity by Equation 9b. The Converse Lyapunov Theorem (Khalil, 2002) then guarantees that the eigenvalues of the Jacobian matrix ∂*f*_η_/∂*x*|_*x* =_*x*__∞_(*v*)_ are strictly inside the unit circle for all *v*. It follows that the matrix


$$\frac{\partial }{\partial x}\left(x-f_{\eta }(v,x))\right)=I-\frac{\partial }{\partial x}f_{\eta }(v,x)$$

is invertible at (*v, x*_∞_(*v*)) for all *v*. We can thus use the implicit function theorem (Rudin, 1976) to find that for each *v*, the solution *x*_∞_(*v*) of the equilibrium equation (Equation 9b) satisfies


$$\frac{\partial }{\partial v}x_{\infty }\left(v\right)={\left(I-\frac{\partial }{\partial x}f_{\eta }\right)}^{-1}\frac{\partial }{\partial v}f_{\eta }$$

with partial derivatives evaluated at (*v, x*_∞_(*v*)). The relation (Equation 29) follows from substituting this expression in Equation 10 and comparing the result to Equation 11.

The fact that Equation 30 is a necessary condition for a bifurcation can be established by applying Schur's complement to express the determinant of the matrix arising from the condition that an eigenvalue of the Jacobian of the linearized model (Equation 1) lies on the unit circle. Finally, Equation 13 and the bifurcation condition (Equation 16) follow directly from taking the real parts of Equations 29 and 30.

## 4 Materials and equipment

### 4.1 Animals

*Cancer borealis* were purchased from Commercial Lobster (Boston, MA) between May 2024 and March 2025 and maintained in tanks containing artificial seawater (Instant Ocean) at 11°C-13°C. Animals were anesthetized on ice for 30 minutes before the stomatogastric nervous system was dissected and pinned in a petri dish coated with Sylgard (Dow Corning) as described previously (Gutierrez and Grashow, 2009). The stomatogastric ganglion (STG) was desheathed to allow intracellular recording. All preparations included the STG, the eosophageal ganglia, and two commissural ganglia. The nervous system was kept in physiological saline composed of 440 mM NaCl, 11 mM KCl, 13 mM CaCl_2_, 26 mM MgCl_2_, 11 mM Trizma base, and 5 mM Maleic acid, pH 7.4-7.5 at 23°C (7.7-7.8 pH at 11°C). All reagents were purchased from Sigma-Aldrich.

### 4.2 Electrophysiology

Extracellular recordings were made by placing 90% Vaseline 10% mineral oil solution wells around the upper lateral ventricular nerve (*lvn*) and stomatogastric nerve (*stn*). Stainless-steel pin electrodes were placed within the *lvn* well to monitor spiking activity and amplified using Model 1700 Differential AC Amplifiers (A-M Systems). Intracellular recordings from the soma of two Gastric Mill (GM) cells were made using two-electrode current clamp with 5-20 MΩ sharp glass microelectrodes filled with 0.6 M K_2_SO_4_ and 20 mM KCl solution. Intracellular signals were amplified with an Axoclamp 900 A amplifier (Molecular Devices, San Jose). Cells were identified by matching intracellular activity with activity on the *lvn*. All amplified signals were digitized using a Digidata 1440 digitizer (Molecular Devices, San Jose) and recorded using pClamp data acquisition software (Molecular Devices, San Jose, version 10.5), sampling frequency of 10 kHz.

After identification of two GM cells, the preparation was disconnected from the descending modulation by adding a solution of 750 mM sucrose and 10^−7^M tetrodotoxin (Sigma) in the *stn* well. Glutamatergic synapses were blocked by adding 10^−5^M picrotoxin (Sigma) to the perfusion solution. For the purposes of real-time data processing the intracellular signals were acquired with NI PCIe-6259 M Series Multifunction DAQ (National Instruments) at 10 kHz sampling frequency, and used in Real-Time eXperiment Interface (RTXI) software version 3.0.

### 4.3 Hardware

All computational work was done in a desktop computer set up with an AMD Ryzen 7 7800X3D 8-Core Processor, 64 Gigabites of RAM, and a NVIDIA GeForce RTX 4090 graphics card with 24 GB of VRAM. Models were trained in the GPU using the Julia library CUDA.jl in conjunction with the module Flux.jl.

## 5 Discussion

What criteria should one use to determine the predictive quality of a data-driven model? Invariably, machine learning and AI approaches use a loss of some kind, and ask how well the model predictions generalize with respect to this loss on unseen data. The choice of loss can be crucial: evaluating the point-wise difference of values on a timeseries can emphasize quantitative variation in a dataset that might be an idiosyncratic feature of *that* dataset. This danger is well known, and places the burden on the experimentalist to measure as broad and ‘representative' a dataset as is feasible.

### 5.1 Deep-learning models for intracellular dynamics

In this paper, we have considered ANN-based models aimed at *quantitative prediction* of intracellular dynamics. Our understanding of quantitative prediction is specific, and helps to narrow the class of data-driven models considered in our work. Throughout this paper, we have implicitly equated the *membrane dynamics* of a neuron with its *system behavior*, a concept borrowed from control theory (Willems, 2007). Following Sepulchre et al. (2018), the system behavior of the membrane is the set of all applied current–membrane voltage trajectory pairs that are consistent with the membrane's biophysics. Concretely, this implies that quantitatively accurate predictions of the membrane dynamics must be consistent with current-clamp and voltage-clamp recordings alike. This view is useful because it unambiguously excludes from consideration many types of data-driven models which can otherwise be said to be predictive (for instance, most integrate-and-fire models, whose suprathreshold behavior is not well-defined).

Following this view, quantitatively predictive models have until recently been almost exclusively “detailed”, “biophysical”, or “conductance-based”, that is, consistent with the mechanistic modeling paradigm established by Hodgkin and Huxley in their seminal work (Hodgkin and Huxley, 1952). However, because conductance-based models are derived from a highly simplified low-dimensional description of neuronal dynamics, their quantitative predictive power is limited. Data-driven models forfeiting quantitative predictions of the membrane dynamics, such as Spike Response Models (Gerstner et al., 2014) and Generalized Linear Models (Pillow and Park, 2016), have repeatedly been shown to beat conductance-based models at predicting particular features of neuronal excitability, most notably spike timing. The ANN-based models considered in this paper can improve on the quantitative prediction of conductance-based models, while keeping the data-driven flexibility of “black-box” statistical models. As we have seen, ANN-based frameworks such as RMMs (Burghi et al., 2025a) further allow biophysical knowledge to inform model architecture. The HCO circuit used in this paper had a known connectivity, allowing us to exploit knowledge of synaptic dynamics to improve on synaptic current predictions. In circuits with unknown connectivity, we have shown that models with minimal biophysical priors can still be trusted to provide reasonable (if not perfectly accurate) predictions of internal circuit currents.

The modeling paradigm studied in this paper prioritizes quantitative analytical predictions over fine biophysical detail, while avoiding the pitfalls of purely statistical models. Recent papers following this paradigm have focused on different aspects of the learning and interpretation problem, but are similar in spirit (Brenner et al., 2022; Durstewitz et al., 2023; Aguiar et al., 2024; Burghi et al., 2025a). Such approaches can in principle be pursued in continuous-time, under the framework of Neural (Chen et al., 2018) and Universal (Rackauckas et al., 2021) ODEs. Interesting questions concerning the potential efficiency gains of continuous-time implementations are left for future work.

### 5.2 Data-driven models can be interpreted using electrophysiology tools

There is a long tradition in electrophysiology of using voltage clamp to study neural excitability via the spectral properties of the neuronal membrane and its currents; see for instance (Mauro et al., 1970; Juusola, 1994; Magnani and Moore, 2011). We have seen that frequency-dependent conductances used to study neural excitability can also be employed to *interpret* a data-driven model which, at first, resembles a black box. This type of analysis was enabled by two model properties: the separation of model states into voltage and internal states, and the contraction of the internal dynamics.

Our results enable relevant extensions of this frequency-domain methodology. First, one could seek to interpret data-driven models with timescale-separated admittances, similarly what is done in Franci et al. (2019) for conductance-based models. The linearity of the internal dynamics of RMMs can readily be exploited for that purpose. Second, instead of analysing admittances neuron by neuron, one could study a data-driven neural circuit by treating its ionic currents as a *multiple-input-multiple-output* system, in which case admittances become matrix-valued (Goodwin and Sin, 1984; Khalil, 2002). Developing these extensions is left for future work.

It is important to distinguish the frequency-dependent conductances obtained from our model from those that could be in principle obtained experimentally with voltage-clamp. For many types of neurons with complex morphology, including the STG neurons used in this paper, voltage-clamp recordings made at a single site (e.g., the soma) leads to distorted recorded currents; see, for instance, (Bar-Yehuda and Korngreen 2008); (Poleg-Polsky and Diamond 2011); (Taylor 2012). Hence, frequency-dependent conductances should be regarded primarily as a tool for model interpretation, rather than for predicting precise experimental I-V relations.

### 5.3 Training methods

While we have emphasized that TF, multiple-shooting and GTF can be understood from a unified perspective, other approaches in the literature can also be associated to these methods. In particular, feed-forward TF as discussed here can be related to the idea of *reservoir computing* (Lukoševičius and Jaeger, 2009). The conceptual difference to the way reservoir computing is usually introduced, is that in data-driven models for intracellular dynamics, the reservoir is an *internal* dynamics, given by Equation 1b, which can be fixed to provide a reservoir of internal states. In neuronal models, learning the “readout” function *h*_θ_ is not the goal but rather a means to learn the forward dynamics (Equation 1). In other words, the task of the reservoir network is to learn the *inverse* dynamics of the neuron or circuit: the dynamics of intrinsic and synaptic ionic currents.

Our paper connects the areas of data-driven models for neural circuit dynamics to contraction theory (Lohmiller and Slotine, 1998) from control engineering. There is a growing literature in systems theory dealing with learning contracting data-driven systems such as recurrent neural networks and equilibrium networks; see, for instance, (Manchester, 2018; Revay and Manchester, 6 11; Pauli et al., 2024).

## Data Availability

The raw data supporting the conclusions of this article can be found in Burghi et al. (2025b), https://doi.org/10.17863/CAM.120457.

## Footnotes

^1^Had we opted for a *passivity-preserving* discretization of the capacitive current (instead of a forward-Euler one), condition (Equation 16) would occur precisely at the boundary of Equation 14.

^2^A positive definite (semi-definite) matrix is a symmetric matrix whose eigenvalues are all positive (non-negative). Negative definite and semi-definite matrices are defined analogously.
